# NMR Assignment of Methyl Groups in Immobilized Proteins Using Multiple-Bond ^13^C Homonuclear Transfers, Proton Detection, and Very Fast MAS

**DOI:** 10.3389/fmolb.2022.828785

**Published:** 2022-03-29

**Authors:** Piotr Paluch, Rafal Augustyniak, Mai-Liis Org, Kalju Vanatalu, Ats Kaldma, Ago Samoson, Jan Stanek

**Affiliations:** ^1^ Faculty of Chemistry, University of Warsaw, Warsaw, Poland; ^2^ Centre of Molecular and Macromolecular Studies, Polish Academy of Sciences, Łódź, Poland; ^3^ Tallin University of Technology, Tallinn, Estonia

**Keywords:** NMR resonance assignment, methyl groups, solid-state NMR, fast MAS, proton detection, TOCSY, isotope labeling/method

## Abstract

In nuclear magnetic resonance spectroscopy of proteins, methyl protons play a particular role as extremely sensitive reporters on dynamics, allosteric effects, and protein–protein interactions, accessible even in high-molecular-weight systems approaching 1 MDa. The notorious issue of their chemical shift assignment is addressed here by a joint use of solid-state ^1^H-detected methods at very fast (nearly 100 kHz) magic-angle spinning, partial deuteration, and high-magnetic fields. The suitability of a series of RF schemes is evaluated for the efficient coherence transfer across entire ^13^C side chains of methyl-containing residues, which is key for establishing connection between methyl and backbone ^1^H resonances. The performance of ten methods for recoupling of either isotropic ^13^C–^13^C scalar or anisotropic dipolar interactions (five variants of TOBSY, FLOPSY, DIPSI, WALTZ, RFDR, and DREAM) is evaluated experimentally at two state-of-the-art magic-angle spinning (55 and 94.5 kHz) and static magnetic field conditions (18.8 and 23.5 T). Model isotopically labeled compounds (alanine and Met-Leu-Phe tripeptide) and ILV-methyl and amide-selectively protonated, and otherwise deuterated chicken α-spectrin SH3 protein are used as convenient reference systems. Spin dynamics simulations in SIMPSON are performed to determine optimal parameters of these RF schemes, up to recently experimentally attained spinning frequencies (200 kHz) and *B*_0_ field strengths (28.2 T). The concept of linearization of ^13^C side chain by appropriate isotope labeling is revisited and showed to significantly increase sensitivity of methyl-to-backbone correlations. A resolution enhancement provided by 4D spectroscopy with non-uniform (sparse) sampling is demonstrated to remove ambiguities in simultaneous resonance assignment of methyl proton and carbon chemical shifts.

## 1 Introduction

For protein studies by nuclear magnetic resonance (NMR), amide and methyl ^1^H resonances are the most commonly exploited. The latter ones are particularly convenient due to the magnetic equivalence of three ^1^H spins (thus threefold sensitivity gain), and enhanced longitudinal relaxation, both caused by fast methyl rotation. In solution NMR, multiple-quantum correlations (HMQC) can be employed to select only the slowly relaxing methyl ^1^H–^13^C coherences (Tugarinov et al., 2003). When combined with extreme ^1^H dilution by deuteration and selective methyl amino acid labeling, the approach allows to study the local dynamics and protein interactions for systems close to MDa molecular weight (MW) (Rosenzweig and Kay 2014; Huang and Kalodimos 2017; Boswell and Latham, 2018).

A prerequisite for the interpretation of NMR data at atomic resolution is a unique, site-specific mapping of chemical shifts to individual atoms. However, conventional resonance assignment strategies for proteins are centered around backbone ^1^H resonances (Sattler, Schleucher, and Griesinger 1999). Chemical shifts of methyl spins, which are peripheral with respect to backbone, are clearly challenging to assign in a systematic way, particularly if detached methyl sites are the only nondeuterated spins. Tailored experiments were developed to correlate methyl to amide frequencies; however, in addition to long coherence transfers involved and thus intrinsically low sensitivity, they require full ^1^H occupancy of (detected) amide sites (Tugarinov and Kay 2003b). The increased proton density is detrimental to ^1^H resolution and sensitivity particularly in large-MW proteins. Alternative strategy relies on correlation to backbone ^13^C′ and ^13^Cα spins (Tugarinov and Kay 2003a), which can be accomplished in the absence of amide ^1^H, but the issue of extended coherence transfer pathway persists.

Mutagenesis is commonly employed to address the methyl assignment issue (Amero et al., 2011). In this approach, single point mutations are introduced, and fingerprint ^1^H–^13^C HMQC spectra are recorded and compared for as many samples as methyl-containing amino acids. The major disadvantages are the labor and cost of isotope-enriched compounds needed to prepare typically tens of samples. Other pitfalls are ambiguities due to overlap of ^1^H–^13^C cross-peaks, or global chemical shift changes induced by mutations (Gorman et al., 2018).

An orthogonal approach is based on the observation of ^1^H–^1^H methyl proximities (NOEs), which can aid the assignment given the presence of the 3D structure determined using other techniques (Gorman et al., 2018). These data are interpreted either by a spectroscopist or, more effectively, by dedicated algorithms intensively developed over the last few years (Schmidt and Güntert 2012, 2013; Pritišanac et al., 2019; Pritišanac, Alderson, and Güntert 2020). The approach depends critically on the NOESY data quality and performs well mostly for methyl ^1^H spins experiencing a dense network of interactions in rigid regions.

Immobilized proteins, such as in amyloid fibrils, sedimented large-MW aggregates, lipid bilayers, or microcrystals, are amenable to solid-state NMR (ssNMR), since this method does not experience high-MW slow-tumbling limitations characteristic of the solution counterpart (Tycko 2011; Miao and Cross 2013; Baker and Baldus 2014; Ladizhansky 2017; Linser 2017; Mandala, Williams, and Hong 2018; van der Wel 2018; Wiegand 2020). The possibility of detection of methyl ^1^H resonances at high resolution in ssNMR was investigated already at low magic-angle spinning (MAS) frequencies (up to 20 kHz), when coupled to high to extreme ^1^H dilution by deuteration (Agarwal et al., 2006; Agarwal and Reif 2008; Asami, Schmieder, and Reif 2010; Asami et al., 2012; Asami and Reif 2013; Mainz et al., 2013). The advent of fast-spinning (above 40 kHz) MAS probes allowed narrow methyl ^1^H lines at significantly higher proton content, either when labile amide ^1^H sites are fully reprotonated (Lewandowski et al., 2011; Linser et al., 2011), or when Ile, Leu, and Val residues are also selectively and nonrandomly (100% CH_3_ or CHD_2_) protonated at methyl sites (Huber et al., 2011; Agarwal et al., 2014; Andreas, et al., 2015a; Kurauskas et al., 2016; Gauto et al., 2019), or when Leu and Val residues are reverse-labeled in an otherwise deuterated matrix (“proton clouds”) (Sinnige et al., 2014). Quite importantly, Schanda and co-workers recently showed that at 55–57 kHz MAS and *B*_0_ = 14.1 T, the ^13^CH_3_ isotopomer yields significantly higher sensitivity compared to ^13^CHD_2_ labeling, and only at a minor loss of ^1^H resolution (Kurauskas et al., 2016). Complete elimination of detrimental sensitivity and resolution effects of strong ^1^H–^1^H dipole interactions by MAS remains a challenge, particularly in methyl-dense protein regions, and would require yet unavailable MAS rates (above 250 kHz) (Kai Xue et al., 2017; K Xue et al., 2018; Kai Xue et al., 2019, Xue et al., 2020). Nevertheless, even in non-deuterated but relatively small proteins, resolved methyl ^1^H–^13^C correlations (of ^1^H linewidths of about 150 Hz) were obtained with 100–111 kHz MAS and high static magnetic fields (23.5 T) (Andreas et al., 2016).

**FIGURE 1:**
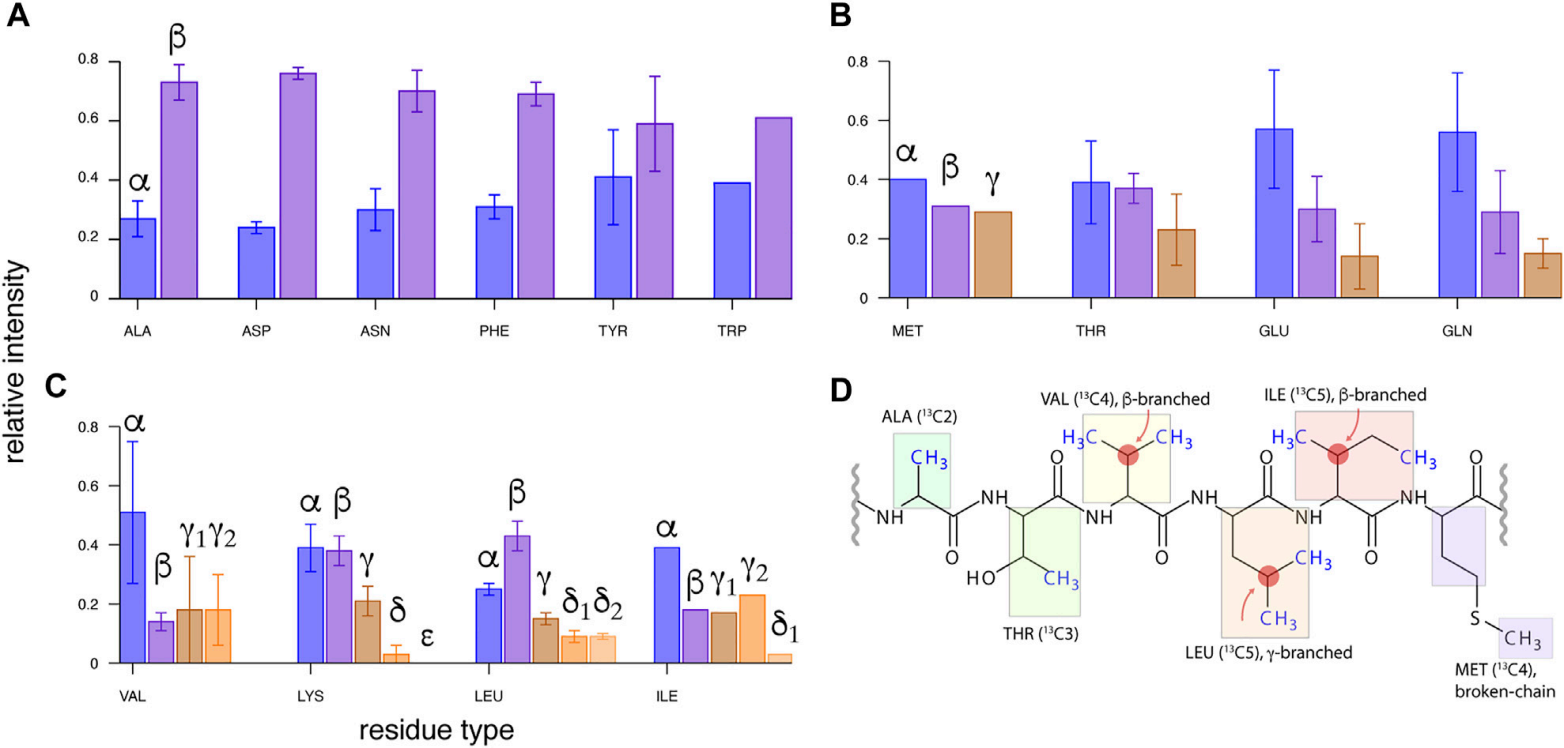
Transfer efficiencies from side-chain ^13^C (β, γ, … ) to ^13^Cα spin for various residues in microcrystalline U-^13^C,^15^N-labeled GB1 protein using ^13^C WALTZ-16 mixing of 15 ms duration, with MAS of ν_R_ = 111 kHz (in a 0.7-mm rotor) and on a 23.5-T spectrometer. Intensity of ^13^C^X^-^13^Cα-^1^Hα cross-peaks in (H)CCH spectrum (Andreas et al., 2016; Stanek et al., 2016) was normalized to a sum of 1.0 for each residue, and averaged within each amino acid type. The data were grouped into **(A)** two- and pseudo-two-, **(B)** three- and pseudo-three-, and **(C)** four- and five-aliphatic ^13^C spin systems (disregarding backbone and side-chain carbonyl and aromatic ^13^C spins). **(D)** Topologies of ^13^C side chains for six amino acid residue types containing a methyl group, from left to right: least to most challenging for methyl resonance assignment.

There are few examples of *de novo* assignment of methyl ^1^H resonances in ssNMR, and frequently the assignment has been aided by either correlations to previously available ^13^C shifts using either dipolar (Agarwal and Reif 2008; Asami and Reif 2012) or scalar-based (Andreas et al., 2015b) (H)CCH-type spectra, or transferred from solution NMR by comparison to relatively uncomplicated ^1^H, ^13^C-CP-HSQC (Huber et al., 2011). In an early work, a strategy was proposed that bases on correlation of methyl resonances to backbone ^13^Cα and C′ spins using (H)CCH-TOBSY experiment, and detection of dilute methyl ^1^H spins, but was found challenging for Leu and Ile residues due to low efficiency of multi-^13^C-bond transfers (Agarwal and Reif 2008). A systematic strategy relying on correlations of side-chain ^13^C to backbone ^15^N and ^1^H, originally proposed by Linser for perdeuterated proteins at slow MAS (25 kHz) (Linser 2011), and more recently at fast MAS (55–60 kHz) with rotor-asynchronous MOCCA mixing (Kulminskaya et al., 2016; Vasa et al., 2018), can be readily adapted for residues selectively 100% reprotonated at methyl sites, but their efficiency with respect to methyl-containing spin systems was not investigated in detail. In the case of non-deuterated (“fully protonated”) proteins, such a long coherence transfer encounters sensitivity limitations, and more practical is a “two-hop” strategy in which side-chain ^1^H and ^13^C resonances are first correlated to alpha ^1^H and ^13^C spins using (H)CCH and H(C)CH experiments (Andreas et al., 2016; Stanek et al., 2016), and subsequently to amide ^1^H and ^15^N shifts using a combination of ^13^Cα–^15^N–^1^H^N^ and ^15^N–^13^Cα–^1^Hα correlations (Zhou et al., 2007; Stanek et al., 2016). Both aforementioned approaches were proposed for a general use (for all amino acid residue types), without a particular emphasis on the methyl assignment. In fact, the literature data on non-deuterated microcrystalline protein GB1 (Andreas et al., 2016) show that WALTZ mixing at MAS rate ν_R_ = 111 kHz and *B*_0_ = 23.5 T leads to an effective ^13^C^x^→^13^Cα transfer only in two-, pseudo-two-, and three-aliphatic ^13^C-spin systems (Figures 1A,B), and becomes problematic for terminal spins in pseudo-three (Glu, Gln)- and larger ^13^C spin systems (Figure 1C). Unfortunately, the latter include valine, leucine, and isoleucine, which additionally suffer from branching of ^13^C chain at β- or γ-positions (Figure 1D). This raises questions of feasibility of methyl resonance assignment under less sensitivity-favorable sample or hardware conditions, and calls for a careful optimization of ^13^C mixing scheme at arising new MAS conditions and *B*_0_ fields.

In this work, we critically assess the efficiency of several ^13^C mixing schemes for methyl ^1^H and ^13^C resonance assignment at fast MAS; determine their optimal range of *B*_0_, MAS frequency, and ^13^C RF strength within and beyond currently available regimes; and explore alternative ^13^C labeling to further boost sensitivity of the approach.

## 2 Materials and Methods

### 2.1 Sample Preparation

2,3-^13^C (99%)-labeled crystalline alanine was purchased from *Eurisotop* (France) and manually packed into a Bruker 1.3-mm MAS rotor.

N-formylated microcrystalline uniformly ^13^C,^15^N-labeled Met-Leu-Phe tripeptide (“fMLF”) was purchased from *Giotto Biotech* (Sesto Fiorentino, Italy) and manually packed into Darklands OÜ 0.81 mm MAS rotor.

The plasmid coding for the Src homology 3 (SH3) domain (965–1,025) of chicken α-spectrin (gene SPTAN1, Uniprot P07751) in a pET3a vector was a kind gift of Dr. T. Schubeis (High Field NMR Centre in Lyon, France). The insert was subcloned into a modified pET28a vector that resulted in a construct including an N-terminal His_6_-tag followed by a SUMO solubility tag. This allowed us to express His_6_-SUMO-SH3 fusion protein.

For preparation of the NMR samples, transformed *Escherichia coli* BL21 (DE3) cells were grown in M9 D_2_O media supplemented with 1 g/L of ^15^NH_4_Cl (*Cortecnet*, France) and 3 g/L of (^2^H,^13^C)-glucose (*Cortecnet*, France) as the sole nitrogen and carbon sources, respectively, following the established procedure (Tugarinov, Kanelis, and Kay 2006). Labeling of the Ile, Leu, and Val side chains was achieved by the addition of the amino acid precursors 1 h prior to the induction with 1 mM IPTG. For Leu and Val residues, we used two kinds of precursors that yield continuous ^13^C chains from methyl (either Cδ or Cγ) to Cα and C′ atoms, provided that ^13^C-enriched glucose is also employed (Goto et al., 1999; Tugarinov and Kay 2003a). The specific labeling patterns and the origin of particular nuclei are shown in Supplementary Figure S1. SH3 sample with branched side chains of leucine and valine residues (hereafter referred to as “ILV-C5” sample) was prepared using 100 mg/L of 1,2,3,4,4′-^13^C-3-^2^H-labeled α-ketoisovaleric-acid (sodium salt, *Eurisotop*, France, catalogue number CDLM-4418-PK) as a Leu and Val precursor. The corresponding sample with linearized ^13^C-side chains of Leu/Val residues (referred to as “ILV-C4” sample) required the addition of 100 mg/L 1,2,3,4-^13^C-3,4′,4′,4′-^2^H-labeled α-ketoisovaleric acid (sodium salt, *Eurisotop*, France, catalogue number CDLM-8100-PK). For both samples, 60 mg/L of 1,2,3,4-^13^C-3,3-^2^H-labeled α-ketobutyric acid (sodium salt, *Eurisotop*, France, catalogue number CDLM-4611-PK) was used as the precursor of isoleucine residues with uniform ^13^C enrichment (Supplementary Figure S1). The cells were grown at 24°C for 18 h after the induction.

The purification protocol of SH3 protein was modified with respect to the original one (Pauli et al., 2000, 2001; Chevelkov et al., 2006). Instead, a standard protocol involving His-trap affinity column and HiLoad 16/60 Superdex 75 gel filtration column (GE Healthcare) was employed. Briefly, we used 50 mM HEPES, 200 mM NaCl, and 1 mM DTT (pH 7.4) supplemented with 20 mM imidazole as a lysis buffer, and the same buffer including additionally 400 mM imidazole was used to elute a protein from the His-trap column. The cleavage of the His_6_-SUMO tag was achieved with a custom-made Ulp1 protease (Reverter and Lima 2009) in a lysis buffer lacking imidazole. The reaction was monitored with the SDS-PAGE and, once completed, the protein was passed through the His-trap column again to remove SUMO as well as His-tagged Ulp1 protease. Cleaved SH3 protein was dialyzed at 4°C overnight against the Superdex 75 running buffer (20 mM citric acid, 150 mM NaCl, pH 3.5), concentrated, and purified on a gel filtration column.

To obtain solid-state NMR protein samples, fractions containing pure SH3 were pooled, concentrated to approximately 10 mg/mL using Vivaspin 3-kDa cutoff centrifugal concentrators (*Sartorius*) and extensively dialyzed against 100 mM (NH_4_)_2_SO_4_ adjusted to pH 3.5 with sulfuric acid. Finally, to crystallize the protein, ammonia water solution was added dropwise to reach pH 7.5. Obtained turbid solutions were stored in a refrigerator (4°C) for approximately a week for a slow buildup of microcrystals. Crystallization efficiency was estimated to 85% by a spectrophotometric measurement of protein concentration decrease in the supernatant. The suspension was mixed with 1 M CuNa_2_EDTA 9:1 v/v (effective *c*_Cu2+_ = 100 mM), and left for impregnation of SH3 crystals with paramagnetic Cu^2+^ ions for 3 days. In the case of the SH3 “ILV-C4” sample, TSP was added to the supernatant at an effective concentration of 10 mM for chemical shift calibration reference. About 2 mg of protein was transferred to a Bruker 1.3-mm MAS rotor by 30 min of ultracentrifugation at an average acceleration of 96,500 *g*. Each Darklands OÜ 0.81-mm MAS rotor was filled with approximately 0.5 mg of protein in five equal parts by stepwise packing in a tailored ultracentrifuge adapter experiencing an average acceleration of 135,000 *g* for 30 min.

### 2.2 Nuclear Magnetic Resonance Spectroscopy

**FIGURE 2:**
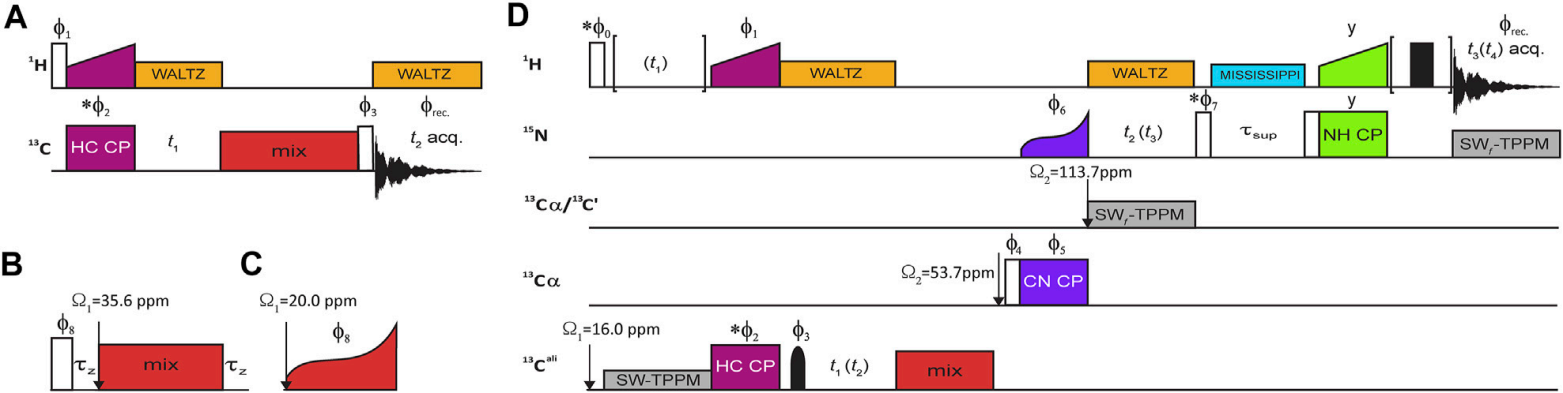
RF irradiation schemes of NMR experiments. **(A)** ^13^C–^13^C 2D correlation experiment with mixing two variants of ^13^C mixing expanded in **(B)** and **(C)**. All schemes act on the ^13^C magnetization preceded and followed by a *z*-filter (τ_z_) **(B)** with the exception of DREAM **(C)**, which operates on the ^13^C single-quantum coherence. **(D)** Pulse diagram for 3D (H)C^MET^(CC)(CA)NH, with the optional evolution period for methyl ^1^H coherences enclosed in brackets (for the 4D variant). The optional ^1^H spin echo immediately before acquisition was only applied for Bruker 1.3-mm probe to eliminate probe ^1^H background signal. Open and solid rectangles represent 90° and 180° high-power pulses, respectively, and a solid bell shape denotes a methyl ^13^C-selective Q3 refocusing pulse (Emsley and Bodenhausen 1992) of 942.7 μs duration and peak RF of ν_1_ = 2.56 kHz. ^13^C mixing is marked in *red*, and cross-polarization transfer steps are labeled and color-coded as *maroon*, *purple*, and *lime green* simultaneous irradiation on pairs of respective channels for H→C, C→N, and N→H transfers. ^1^H low-power WALTZ (Shaka, Keeler, and Freeman 1983) decoupling is marked in *orange*, while heteronuclear (^13^C or ^15^N) frequency-swept TPPM decoupling (Bennett et al., 1995) is marked in *gray*. MISSISSPPI solvent suppression (Zhou and Rienstra 2008) is marked in *cyan*. Changes in ^13^C offset (Ω) are denoted with vertical arrows. Unless indicated otherwise, all pulse phases are set to *x*. The following phase cycling was employed: *ϕ*_1_ = *y*, *ϕ*_2_ = *y*, *ϕ*_3_ = {2 (*x*), 2 (−*x*), 2 (*y*), 2 (−*y*)}, *ϕ*_8_ = {*x*, −*x*}, ϕ_rec._ = {*x,* −*x*, −*x*, *x, y,* −*y,* −*y, y*} for 2D experiment with inset **(B)**; *ϕ*_1_ = {*y,* −*y*}, *ϕ*_2_ = {2 (*x*), 2 (−*x*)}*, ϕ*_3_ = {4 (*x*), 4 (−*x*)}, *ϕ*_8_ = {2 (*x*)*,* 2 (−*x*)}, ϕ_rec._ = {*x,* −*x, x,* −*x*} for 2D DREAM experiment (A + C); *ϕ*_1_ = {4 (*y*), 4 (−*y*)}, *ϕ*_2_ = *x*, *ϕ*_3_ = {*x*, *x*, *y*, *y*}, *ϕ*_4_ = *y*, *ϕ*_5_ = {8 (*x*), 8 −*x*)}, *ϕ*_6_ = {*y, –y*}, *ϕ*_7_ = *x*, *ϕ*_8_ = -*y*, ϕ_rec._ = {*x, –x, –x, x, –x, x, x, –x, –x, x, –x, x, –x, –x, x*} for 3D experiment (D) with recoupling block (B); and *ϕ*_1_ = {8 (*y*), 8 (−*y*)}, *ϕ*_2_ = {4 (*x*), 4 (−*x*) }, *ϕ*_3_ = {2 (*x*), 2 (*y*), 2 (−*x*), 2 (−*y*)}, *ϕ*_4_ = *x*, *ϕ*_5_ = *x*, *ϕ*_6_ = {*y*, −*y*}, *ϕ*_7_ = {*x*}, *ϕ*_8_ = {4 (x), 4 (– *x*)}, ϕ_rec._ = {*x, –x, –x, x, –x, x, x, –x, –x, x, –x, x, –x, –x, x*} for 3D DREAM experiment (D + C). Pulse with phase marked with an asterisk are involved in quadrature detection according to States-TPPI procedure.

The NMR ^13^C–^13^C 2D correlation experiments on model compounds (alanine and fMLF) were performed using a standard RF irradiation scheme shown in Figure 2A. A collection of homonuclear mixing schemes was employed as detailed below. For each case, a series of 2D spectra with gradually incremented mixing time was acquired, in the range suggested by literature and spin dynamics simulations, if permitted by probe RF circuity.

The experiments for site-specific assignment of methyl ^13^C and ^1^H resonances in SH3 protein that provide correlations to backbone amide ^15^N and ^1^H chemical shifts [3D (H)C(CC)(CA)NH] were straightforwardly adopted from the literature (Linser 2011) and are shown in Figure 2B. Despite long methyl ^1^H transverse relaxation times observed, the experiments employ ^1^H–^13^C cross-polarization (CP) instead of INEPT-type transfer, since the latter would suffer from inefficiency of conversion of anti- to in-phase ^13^C coherence caused by concurrent evolution of two (passive) ^1^ *J*_CH_ couplings in CH_3_ moieties. Implementations of presented experiments for Bruker spectrometers are freely available from the community-based repository Zenodo as detailed below.

For the site-specific evaluation of experimental performance with various ^13^C mixing schemes, resolution provided by 3D spectra was required, preventing acquisition of mixing time series for SH3 protein. Optimal mixing times were, however, possible to determine using first-increment 1D optimizations prior to 3D data acquisition.

The series of 2D ^13^C–^13^C correlation experiments on 2,3-^13^C-alanine was carried out on a Bruker Avance III spectrometer operating at ^1^H and ^13^C frequency of 600.1 and 150.9 MHz, respectively, equipped with a Bruker 1.3-mm H/C/N MAS probehead. The sample has been spun at 55,555 ± 10 Hz and controlled by a Bruker MAS-II unit, without temperature stabilization. ^1^H, ^13^C pulses and power during ^1^H to ^13^C cross-polarization have been carefully calibrated prior to experiments. Detailed information on pulse lengths, RF amplitude, spectral windows, etc. is provided in Supplementary Table S1.

2D experiments on ^13^C,^15^N-fMLF tripeptide and 3D and 4D experiments on two SH3 protein samples were performed on a Bruker Avance III HD spectrometer operating at a ^1^H, ^13^C, and ^15^N frequency of 799.7, 201.1, and 81.0 MHz, respectively, equipped with a 0.81-mm H/C/N/D MAS probehead developed by Ago Samoson’s group (Darklands OÜ, Estonia). NMR data were acquired at two MAS frequencies, ν_R_ = 55.5 and 94.5 kHz (98 kHz for fMLF).

3D experiments on “ILV-C5”-labeled SH3 protein were also performed on a Bruker NEO 23.5-T spectrometer (in CRMN Lyon, France) equipped with a Bruker 1.3-mm H/C/N/D MAS probe, and MAS-III and BCU-II spinning and temperature stabilization units.

For the experiments on ^13^C,^15^N-fMLF magic angle setting has been set using KBr sample prior to the actual series. In this case, no temperature stabilization device was used. ^1^H and ^13^C 90° pulse lengths were carefully calibrated using 1D ^13^C-detected CP experiment.

Prior to experiments on SH3 samples, a careful magnet shimming was performed to maximize reliability of linewidth measurements. The classical shimming protocol employing adamantane sample is very time-consuming in 0.81-mm MAS rotors; thus, a sample of silicon grease was used first, leading to full-width at half-height (FWHH) of ^1^H line of 16 Hz at 50 kHz MAS. Subsequently, shim currents were refined using adamantane sample resulting in an FWHH of the low-field ^13^C resonance of 1.1 Hz at 50 kHz MAS under low-power ^1^H decoupling.

Magic angle was finely adjusted directly on the protein sample at the target MAS frequency by maximizing the intensity of the ^1^H signal in the (H)NH 1D experiment followed by a 5-ms ^1^H spin echo. Sample temperature was stabilized to 20°C using Darklands OÜ VT controller. Thermocouple readout was calibrated to sample temperature by measurement of the ^207^Pb chemical shift of Pb(NO_3_)_2_ at the exactly same cooling gas flow, spinning speed, and thermocouple target temperature. ^1^H, ^15^N 90° pulse lengths have been carefully calibrated for each sample and spinning speed using the (H)NH experiment. ^13^C 90° pulse length was calibrated using the (H)CONH experiment. RF amplitude of hard and soft pulses, as well as of decoupling and recoupling schemes was automatically calculated in a pulse program based on widths and powers of reference high-power 90° pulses. ^1^H–^13^C, ^13^C–^15^N, and ^15^N–^1^H cross-polarization power was optimized directly using the first increment of the (H)C(DIPSI)(CA)NH experiment, and propagated to all 3D (and 4D) experiments at each sample and spinning condition. Fast recycling (0.3 or 0.4 s interscan delay) was applied to improve sensitivity, taking advantage of longitudinal ^1^H relaxation enhancement by paramagnetic Cu^2+^ ions (Ganapathy et al., 1981; Wickramasinghe et al., 2007, 2009). Details on NMR data acquisition for fMLF tripeptide and SH3 protein are provided in Supplementary Tables S1, S3, respectively.

*B*_0_ was not stabilized in either case, but the field drift was monitored using 1D ^1^H spectra between experiments and had a negligible effect on data (e.g., a total 11 Hz ^1^H downfield drift over 10 days on an 18.8-T spectrometer for “ILV-C5” SH3 sample at 94.5 kHz MAS). The stability of CP conditions was monitored using 1D (H)(CA)(N)H and (H)(C)(DIPSI)(CA)(N)H (for proteins) or ^13^C-detected 1D CP (for fMLF) experiments.

2D and 3D data were Fourier processed in Bruker TopSpin using parameters reported in Supplementary Tables S2, S4. The non-uniformly sampled 4D data were converted using in-house written script *bruk2ssa* (courtesy of M. Górka), and processed using signal separation algorithm for distortion-free spectral reconstruction (Stanek, Augustyniak, and Koźmiński 2012). All spectra were analyzed in NMRFAM-Sparky (Lee, Tonelli, and Markley 2015).

### 2.3 Homonuclear ^13^C Mixing Schemes

The key element determining the performance of experiments shown in Figure 2 is ^13^C mixing. For this comparative study, we have made a selection of literature RF designs based on the following criteria: (1) ^13^C should occur primarily between bonded ^13^C spins by recoupling of ^13^C–^13^C scalar (*J*) or dipolar (*D*) interactions, (2) RF requirements are acceptable for a typical “fast” MAS probe, and (3) they pose no danger to the hydration of fragile protein samples, e.g., by an extended period of high-power ^1^H irradiation. For the last reason, we did not consider second-order recoupling schemes suitable for fast MAS, such as MIRROR (Scholz et al., 2008), PARIS (Weingarth et al., 2009), SHANGHAI (Hu et al., 2011), or CORD/CORD-RFDR (Hou et al., 2013; Lu et al., 2015). In the presence of relaxation, the efficiency of these sequences deteriorates with faster MAS, concomitantly to increasing efficiency of averaging of H–H dipolar interactions. Protein deuteration, which is highly recommended for resolution in large proteins, is also clearly incompatible with second-order recoupling mechanism (De Paëpe, Lewandowski, and Griffin 2008) as it prohibitively dilutes the proton interaction network.

#### 2.3.1 TOBSY

**FIGURE 3:**
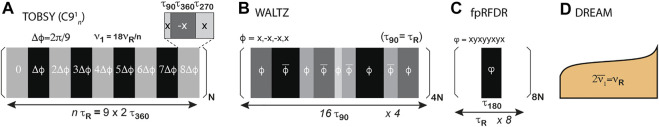
RF schemes of ^13^C mixings compared in this study. **(A)** TOBSY C9^1^_*n*_ variants with variable *n*. **(B)** WALTZ-16, a representative of TOCSY class. The primitive element is composed of phase-alternated 90, 180, 270, and 360° pulses, depicted as rectangles color-coded from *light gray* to *black*. **(C,D)** Transfer schemes primarily recoupling ^13^C dipolar interactions: **(C)** finite-pulse RFDR and **(D)** DREAM.

TOBSY was designed based on average Hamiltonian theory (AHT) and symmetry properties of particular interactions (Levitt 2007), imposing that chemical shift anisotropy (CSA), isotropic chemical shifts (CS), and dipolar interaction terms are suppressed in the zeroth- and first-order Hamiltonians, and the zeroth-order term stems only from the isotropic scalar interaction (Baldus and Meier 1996; Hardy, Verel, and Meier 2001). In a general design denoted CN^ν^_*n*_ (Edén and Levitt 1999), a primitive multi-pulse block C is repeated N times over *n* rotor cycles with the gradual phase incrementation in Δϕ = 2πν/N steps (Figure 3A). Low-power TOBSY schemes suitable for fast MAS employ POST primitive block (90_ϕ_360_ϕ+π_270_ϕ_), and use *N* = 9, ν = 1, and *n* = 9*p* ± 3 (where *p* is an integer), e.g., C9^1^_21_, C9^1^_24_, and C9^1^_30_, to retain only assumed interaction (Tan et al., 2018). The schemes with particular *n* differ in robustness to dipolar C–H interactions and susceptibility to CSA (in higher-order AHT terms). TOBSY requires an RF strength of ν_1_ = 2N/*n* ν_R_ and thus is readily applicable in typical fast MAS probes for *n* > 18. Here, we employed sequences with *n* = 24, 30, 33, 39, and 48, with mixing time either varied between 0 and 50 ms (in 2D series) or optimized for maximum transfer (in 3D experiments).

#### 2.3.2 TOCSY

Isotropic mixing schemes developed for ^13^C mixing in solution NMR (Bax, Clore, and Gronenborn 1990; Kay et al., 1993), hereafter referred to using a general term TOCSY, retain only the Hamiltonian term associated with scalar (*J*) interaction between ^13^C nuclei (in the presence of the overall tumbling). For solids under MAS, all isotropic interactions are preserved; thus, these RF schemes induce the evolution of *J*_*CC*_ interactions while suppressing the evolution of *J*_HC_ and isotropic ^13^C chemical shifts. The proper treatment of anisotropic interactions is not ensured at all; however, at sufficiently fast MAS, their contribution is *supposedly* small. Simple arguments advocated for the use of WALTZ-16 (Shaka, Keeler, and Freeman 1983) mixing synchronized with rotation (τ_90_ = τ_R_; Figure 3B), and basic properties were verified using 2-^13^C-spin SIMPSON simulations (Andreas et al., 2016). Classical TOCSY mixing schemes that are robust with respect to large chemical shift offsets, namely, DIPSI-3 (Shaka, Lee, and Pines 1988) and FLOPSY-16 (Kadkhodaie et al., 1991), were also employed here. In solution NMR, TOCSY designs are ranked according to the figure of merit, i.e., the bandwidth with respect to applied RF strength, but such a ranking is of limited relevance here since MAS probes can easily generate sufficient ν_1_. Although not required, we retained rotor synchronization (ν_1_ = ¼ ν_R_) in experiments for three selected TOCSY sequences, but tested other RF conditions in spin dynamics simulations. Mixing time was varied (for alanine and fMLF) or optimized in 1D experiments (for proteins) in steps of 188.448, 217.32, and 96.0 τ_R_ (assuming τ_R_ = τ^C^_90_) for FLOPSY, DIPSI, and WALTZ, respectively.

#### 2.3.3 RFDR

Finite-pulse ^13^C RFDR (Bennett et al., 1998) recouples dipolar ^13^C interactions in the first-order average Hamiltonian by the application of high-power π pulse each rotor cycle (Figure 3C). Although the sequence is known to suffer from dipolar truncation (Griffin 1998), this is not necessarily a disadvantage for intraresidue ^13^C transfers. RFDR requires ν_1_ > ½ ν_R_ for pulses partially covering the mixing time; in practice, ν_1_ on the order of ν_R_ is preferred. The ratio of τ_180_/τ_R_ determines the scaling factor of dipolar interaction (nominally of about 2 kHz for a bonded ^13^C–^13^C spin pair), and in this study, we used ν_1_ = 150 (for fMLF) and 100 kHz (otherwise). The following is the phase cycle (xy8) of the π pulse: *x*, *y*, *x*, *y*, *y*, *x*, *y*, *x*, as suggested previously (Shen et al., 2012). Mixing time was optimized in steps of multiples of 8τ_R_ between 0 and 50 ms.

#### 2.3.4 DREAM

DREAM recouples dipolar ^13^C interactions in the first-order average Hamiltonian using an adiabatic pulse for introducing HORROR condition (ν_1_ = ½ ν_R_, Figure 3D) (Verel et al., 1998; Verel, Ernst, and Meier 2001). DREAM is commonly employed with slow (Pauli et al., 2001) and fast MAS (Penzel et al., 2015) to trigger ^13^Cα→^13^Cβ or ^13^C′→^13^Cα coherence transfer, for which RF and offset conditions can be straightforwardly determined. Transfers in multi-spin systems were studied and conditions were optimized at “slow” MAS (Westfeld et al., 2012); however, the proper order of HORROR conditions cannot in general be satisfied due to characteristic chemical shifts in ^13^C side chains. Additionally, by its nature, DREAM cross-peak intensity is negative with respect to the origin coherence, which potentially causes destructive interferences within a spin system or due to spectral overlaps. The RF shape was defined as usual, $v\left(t\right)=\bar{v_{1}}+d^{eff}\text{tan}\left(\frac{2}{\tau }{\text{tan}}^{-1}\;\left(\Delta /d^{eff}\right)\left(t-\tau /2\right)\right)$ , where $d^{eff}$ , Δ, τ, and $\bar{v_{1}}$ denote the effective dipolar coupling (averaged over crystal orientations and decreased due to dynamics), modulation depth, mixing time, and average RF strength, respectively. Directionality of the transfer was selected here with increasing RF over mixing time. We employed the parameters recommended for DREAM adiabatic RF modulation at fast MAS, namely, the modulation depth of 1/5 ν_R_, dipolar C–C coupling of 1 kHz, and average RF ${\bar{v}}_{1}$ to nominally ½ ν_R_, which potentially allows DREAM to cover the entire aliphatic ^13^C band at typical high fields (e.g., 18.8 T). In addition to the mixing time (varied up to 10 or 25 ms for alanine, or fMLF and SH3, respectively), ^13^C offset for DREAM was also optimized for best ^13^C^met^→^13^Cα transfer in the 2D series for alanine and fMLF, and using 1D first-FID of sequence shown in Figure 2D.

### 2.4 Spin Dynamics Simulations Using SIMPSON

Spin dynamics simulations have been performed using SIMPSON (version 4.0.0c) (Bak, Rasmussen, and Nielsen 2011; Tošner et al., 2014). Powder averaging has been performed with 1,848 {α, β, γ} Euler angles that describe the orientation of the molecule in the rotor frame. A total of 168 {α, β} angle pairs have been selected using REPULSION algorithms (Bak and Nielsen 1997) and 11 γ angles have been regularly sampled from 0 to 360°. Spin systems have been generated using the SIMMOL package (Bak et al., 2002).

For simulations in the case of a model four-spin system (Hα–Cα–Cβ–Hβ), dipolar spin couplings have been generated based on distances in L-alanine structure (ref. code LALNIN61 in CCS database), and only α and β carbons and protons have been considered. ^1^ *J*_CC_ and ^1^ *J*_HC_ couplings have been set to 33 and 145 Hz, respectively. In addition, only one proton from the CH_3_ group has been considered with the H–C dipolar coupling value reduced by three due to fast rotation around the C–C axis. Other relevant parameters of the spin system are reported in the Supplementary Material.

For simulation in the case of C_6_ and C_5_ spin systems, dipolar couplings have been calculated for the geometry of leucine-8 in the X-ray structure of chicken SH3 protein (PDB 1SHG). Experimentally determined chemical shifts in fMLF have been assumed, and CSA parameters were calculated in Gaussian. ^1^ *J*_CC_ couplings were set to 50 Hz for the ^13^Cα–^13^C′ pair and to 33 Hz between aliphatic ^13^C spins. ^2^ *J*_CC_ was set to 3 Hz, and longer-distance *J* couplings were neglected. Examples of SIMPSON input files are provided in the Supplementary Material.

## 3 Results and Discussion

### 3.1 Homonuclear Mixing in Model Two-^13^C Spin Systems

Efficiency of ^13^C mixing varies in general with MAS frequency ν_R_, RF strength ν_1_ (if not imposed by ν_R_), *B*_0_ field (through scaling of isotropic chemical shift differences and CSA), presence of ^1^H–^13^C interactions, and extent of dynamics as well as other design-specific parameters detailed above. To mitigate the complexity of the problem, one usually refers to model two-^13^C spin systems, such as, e.g., in the acetate anion. Here, we used 2,3-^13^C_2_,^15^N-labeled crystalline alanine as a closer analogue of a protein residue, additionally possessing a methyl group, and avoiding interference with large CSA of the (unlabeled) carbonyl carbon. The efficiency of ^13^Cβ→^13^Cα transfer was quantified by observation of cross-peak intensity in a τ_mix_ series of 2D ^13^C–^13^C spectra at fast MAS (ν_R_ = 55.5 kHz) and moderate *B*_0_ field (14.1 T).

**FIGURE 4:**
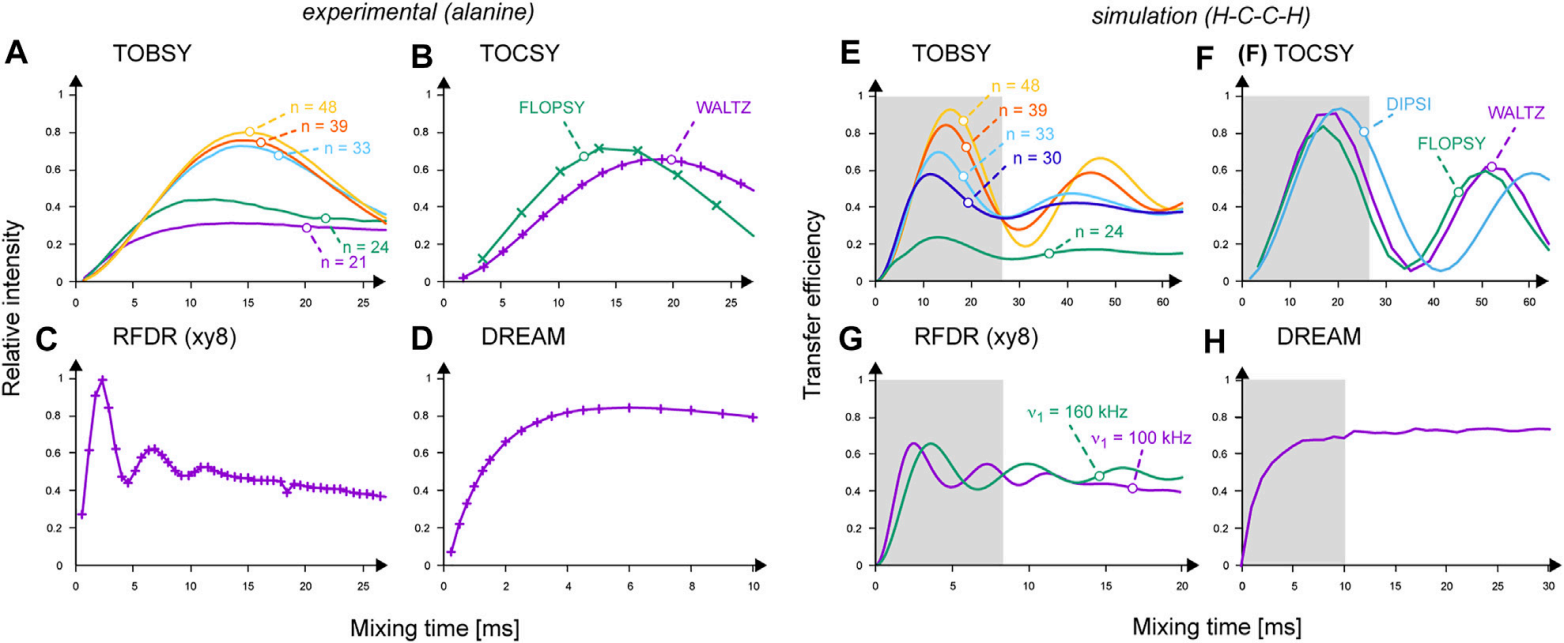
**(A–D)** Performance of ^13^C mixing schemes observed experimentally in ^13^C–^13^C 2D spectra on 2,3-^13^C_2_-labeled alanine: **(A)** TOBSY C9^1^_*n*_ with *n* = 21, 24, 33, 39, and 48 are labeled and color-coded as *purple*, *green*, *cyan*, *red*, and *orange* curves, respectively; **(B)** FLOPSY-16 (in *green*) and WALTZ-16 (in *purple*); **(C)** RFDR at ν_1_ = 100 kHz; **(D)** DREAM. Cross-peak intensities were normalized to the most intense point in the RFDR series. Experimental points are connected with lines for eye guidance only. **(E–H)** Buildup of transferred ^13^C magnetization (absolute transfer efficiency) in a two ^13^C- and two ^1^H-spin system simulated in SIMPSON. **(E)** TOBSY C9^1^_*n*_ with *n* = 24, 30, 33, 39, and 48 are labeled and color-coded as *green*, *dark blue*, *cyan*, *red*, and *orange* curves, respectively; **(F)** FLOPSY-16 (in *green*), DIPSI-3 (in *light blue*) and WALTZ-16 (in *purple*); **(G)** RFDR at ν_1_ = 100 (in *purple*) and 160 kHz (in *green*); **(H)** DREAM. Gray boxes indicate the mixing time ranges sampled experimentally on alanine.

The results shown in Figure 4 confirm the high efficiency of selected RF designs. Also, fundamental differences between them exhibit the following: TOBSY and TOCSY sequences recouple *J*_CC_ in a time close to 1/(2J) (scaling factors might apply to the effective coupling constant, e.g., for FLOPSY). RFDR initially shows rapid oscillations with frequency related to a scaled dipolar coupling (*D*_CC_ ≈ 2 kHz), stabilizes at approximately τ_mix_ = 5–10 ms, and then decays due to π pulse imperfections and incoherent effects. DREAM shows a steady buildup, reaches a plateau at τ_mix_ ≈ 5 ms, and then decays slowly mostly due to ^13^C T_1ρ_-like relaxation. The results are generally consistent with the literature, also confirming the increasing robustness of TOBSY C9^1^_*n*_ to *D*_HC_ interactions with increasing *n* (Figure 4A) (Tan et al., 2018). Although RFDR experimentally showed highest performance, observed differences between considered schemes are rather minor.

Spin dynamics simulations are instrumental for the in-depth understanding of complex RF schemes applied under new MAS conditions. Here, we resorted to SIMPSON as a generally agreed simulation platform (Tošner et al., 2014), and attempted first to reproduce experimental results. We modeled alanine using a minimal system consisting of two ^13^C and ^1^H spins as described above. Results shown in Figures 4E–H reproduce well the experimental buildup curves, optimal mixing times, and scaling factors of recoupled interactions. Differences in efficiency between TOBSY C9^1^_*n*_ sequences with *n* = 48, 39, 33, and 24 (Figure 4E) are more pronounced than in the experiment (Figure 4A). C9^1^_48_ allows for an almost complete transfer for τ_mix_ ≈ 16 ms. For longer mixing times, recoupling of higher-order AHT terms occurs (mostly the cross-terms between ^13^C isotropic and anisotropic chemical shifts, and *D*_HC_ interactions), leading to coherence dephasing and decreased intensity of subsequent maximums. Somewhat surprisingly, all TOCSY sequences perform excellently (Figure 4F), despite no deliberate treatment of anisotropic interactions. RFDR performance is relatively worse than in the experiment, and equalizes ^13^C magnetization between both coupled ^13^C spins. Supposedly, incoherent effects and *B*_1_ field inhomogeneity in the MAS coil affect RFDR to the smallest extent among the considered mixing types. In simulations, DREAM does not show an optimum, but a steadily increasing coherence transfer.

Having validated the simulation platform, we attempted to differentiate ^13^C mixing schemes with respect to destructive interferences (cross-terms) with interactions that are field-dependent (isotropic and anisotropic CS), and proton-content-dependent (*D*_H-C_ interactions). All combinations of selectively “activated” interactions were probed, and the effects of most relevant cross-terms are summarized in Supplementary Figure S2. We confirmed that TOBSY C9^1^_n_ sequences with *n* > 30 are quite robust to *D*_HC_, with (*J*_CC_, *D*_HC_), (*D*_CC_, *D*_HC_), and (*D*_CC_, CSA) being the primary cross-terms responsible for non-ideal performance. We additionally confirmed that TOBSY is robust with respect to an increasing chemical shift difference (or *B*_0_ field), e.g., up to ΔΩ = 15 kHz for C9^1^_24_ at ν_R_ = 55 kHz (and v_1_ = 2/3 ν_R_). Among TOCSY sequences, FLOPSY appeared to be most susceptible to (*J*_CC_, *D*_HC_) interferences (or, in other words, to the presence of protons). Also, all TOCSY designs are less affected by (*D*_CC_, *D*_HC_) terms than TOBSY and very robust to CSA (Supplementary Figure S2). Thus, WALTZ and DIPSI are good candidates for high *B*_0_ field measurements, and on systems with high proton density. Within the initial buildup, RFDR is extremely robust to all interaction interferences, and in fact even enhanced by recoupling of *J*_CC_ interactions.

Additionally, we verified the susceptibility of TOBSY and TOCSY sequences to *B*_1_ field inhomogeneity, which can be substantial in MAS RF coils [as large as 20% in the active volume (Tošner et al., 2018)]. As shown in Supplementary Figure S3, TOBSY sequences only tolerate maximum 5% RF deviations (or miscalibration), and TOCSY designs, DIPSI in particular, are significantly more robust with this respect.

However, multiple limitations of spin dynamics simulations must be considered when their results are interpreted quantitatively: (1) no incoherent effects (relaxation) are included; (2) structural variability of dipolar interactions, chemical shifts, and CSA are tedious to replicate; and (3) pulse transients, limited short-term rotation stability, and other experimental deficiencies are neglected.

### 3.2 Homonuclear ^13^C Transfer Across Multi-Spin Side Chains in Model Systems

**FIGURE 5:**
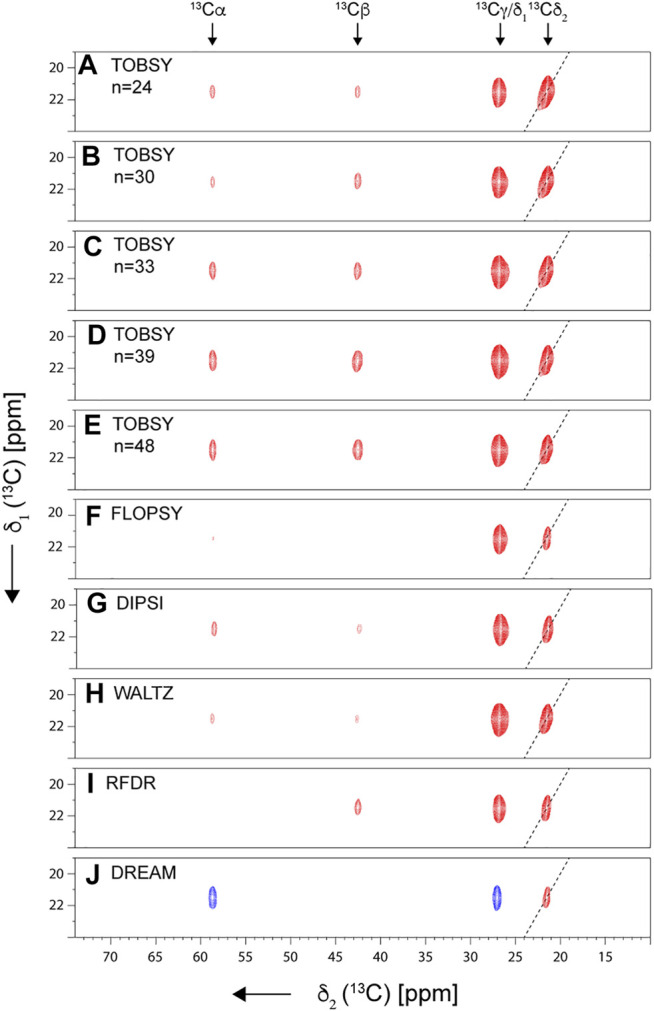
Strips from 2D ^13^C–^13^C spectra recorded for fMLF at ν_R_ = 55.5 kHz on an 18.8-T spectrometer. Presented are diagonal and cross-peaks originating from Leu ^13^Cδ_1_ magnetization (prior to ^13^C mixing). Spectrum diagonal is indicated with a dashed line. Spectra with a mixing time optimal for Cδ_1_→ Cα transfer are shown: 34.6, 32.0, 21.4, 25.3, and 36.3 ms for TOBSY C9^1^_*n*_, with *n* = 24, 30, 33, 39, and 48, respectively **(A–E)**; 23.7, 27.4, and 20.7 for FLOPSY-16 **(F)**, DIPSI-3 **(G)**, and WALTZ-16 **(H)**, respectively; and 11.2 and 14 ms for RFDR (ν_1_ = 150 kHz) **(I)** and DREAM **(J)**, respectively.

Subsequently, we performed an analogous series of 2D ^13^C–^13^C correlation spectra on a sample of crystalline tripeptide fMLF, where the leucine residue serves as a realistic and convenient reference multi-spin system containing a methyl group. Spectral excerpts in Figure 5 demonstrate that, while one-bond transfers remain effective in all cases, efficiencies of multi-bond ones are low and differ dramatically between particular RF schemes (see Supplementary Figure S4 for mixing time dependencies). In contrast to the case of alanine, RFDR performs worst in this respect, emphasizing the limited utility of simplistic two-^13^C spin systems for evaluation of ^13^C mixing performance.

It was noted previously in both solution (Tugarinov and Kay 2003a) and solid-state NMR (Agarwal and Reif 2008) that a combination of a branched ^13^C chain, large number of ^13^C spins, and large chemical shift differences makes the leucine spin system extremely challenging for any ^13^C mixing. In the following, we will deliberately focus on the most demanding Cδ_1_→Cα transfer as the sensitivity-limiting step, regardless of which side-chain assignment strategy mentioned above [the one-step side-chain to backbone, (H)C(CC)(CA)NH, or the two-step approach using (H)CCH and (H)NCAHA spectra] is employed. (Another potentially relevant transfer Cδ_1_→C′ is expected to be even more problematic due to a large CSA and distinct chemical shift.)

**FIGURE 6:**
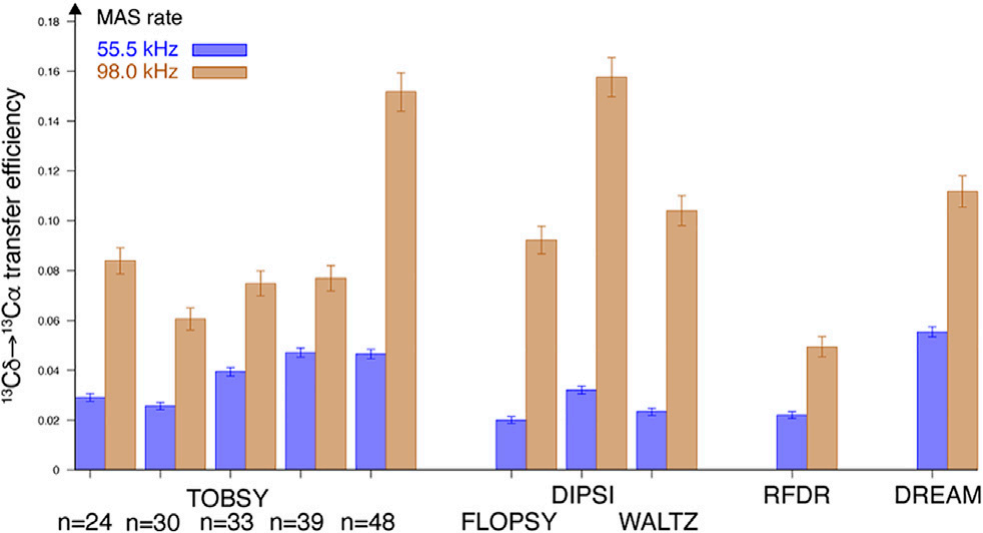
Maximum absolute efficiency of Cδ_1_ → Cα magnetization transfer observed in leucine residue of fMLF at *B*_0_ = 18.8 T field and at ν_R_ = 55.5 (*blue* bars, left in each pair) and 98 kHz (*tan* bars, right in each pair).

The entire experimental series was repeated at very fast spinning conditions (ν_R_ = 98 kHz) to probe sensitivity of selected mixing schemes to coherent and incoherent effects of dipolar C–H and H–H interactions. Absolute efficiencies of Cδ_1_→Cα transfer were quantified as described in Supplementary Material, and presented in Figure 6. Strikingly, all RF schemes greatly benefit from increased MAS rate (up to a factor of 4.5–5 for three TOCSY representatives). At ν_R_ = 55.5 kHz DREAM performs best, and the most efficient scheme at ν_R_ = 98 kHz is DIPSI, with TOBSY C9^1^_48_ being close to the leaders at both MAS frequencies. In both cases, RFDR and TOBSY C9^1^_n_ with *n* < 30 remain evident outliers.

Given such a dramatic response to MAS frequency, we attempted to verify capabilities of spin dynamics simulation for reproducing the behavior of a complex, but extremely useful leucine spin system, and possibly extrapolate it to different spinning and field conditions. Unfortunately, despite intense optimization of SIMPSON routines (e.g., the reuse of propagators) and the use of the state-of-the-art high-performance clusters, inclusion of any meaningful number of ^1^H spins (*L* ≈ 5–6) turned out unfeasible due to the exponential scaling of required computational resources (4^*L*^). Nevertheless, many features of the transfers were quite well reproduced in the exclusively ^13^C 6-spin system, in particular (1) the optimal Cδ_1_ → Cα mixing times (Supplementary Figure S5), (2) the shape of build-up curves for individual transfers (Supplementary Figure S6), and (3) poor performance of RFDR (≈5%) at both spinning speeds. The results also corroborate TOBSY C9^1^_48_ and DIPSI as the performance leaders at ν_R_ ≈ 100 kHz (Supplementary Figure S7). The exact quantification of DREAM poses difficulties due to the absence of an optimum in simulations, but efficiency at τ_mix_ = 25 ms, as used in the experiments, places this mixing scheme closer to the best than to mediocre designs.

The absence of protons in the spin system used in simulations resulted in almost identical performance of all TOBSY schemes at ν_R_ = 100 kHz, which clearly disagrees with the experiments. Likely for the same reason, the experimentally observed relative order of performance of TOCSY sequences was not reproduced. To some extent, one can still rely on the analysis of cross-term importance for four spin system (Supplementary Figure S2) to predict the impact of protons also in leucine residues. However, absolute efficiencies predicted in simulations (Supplementary Figure S7) and observed experimentally (Figure 6) show discrepancies from roughly 25% (for TOBSY C9^1^_48_ and DIPSI) to more than 100% (e.g., for FLOPSY and WALTZ) at ν_R_ = 98–100 kHz, and even larger ones at the lower spinning speed (ν_R_ = 55.5 kHz). These disagreements arise not only due to the (unaccounted) coherent effects of ^1^H–^13^C interactions, but also due to ^13^C relaxation, suggesting the ultimate need of experimental verification for a quantitative comparison at specific ν_R_ and *B*_0_.

**FIGURE 7:**
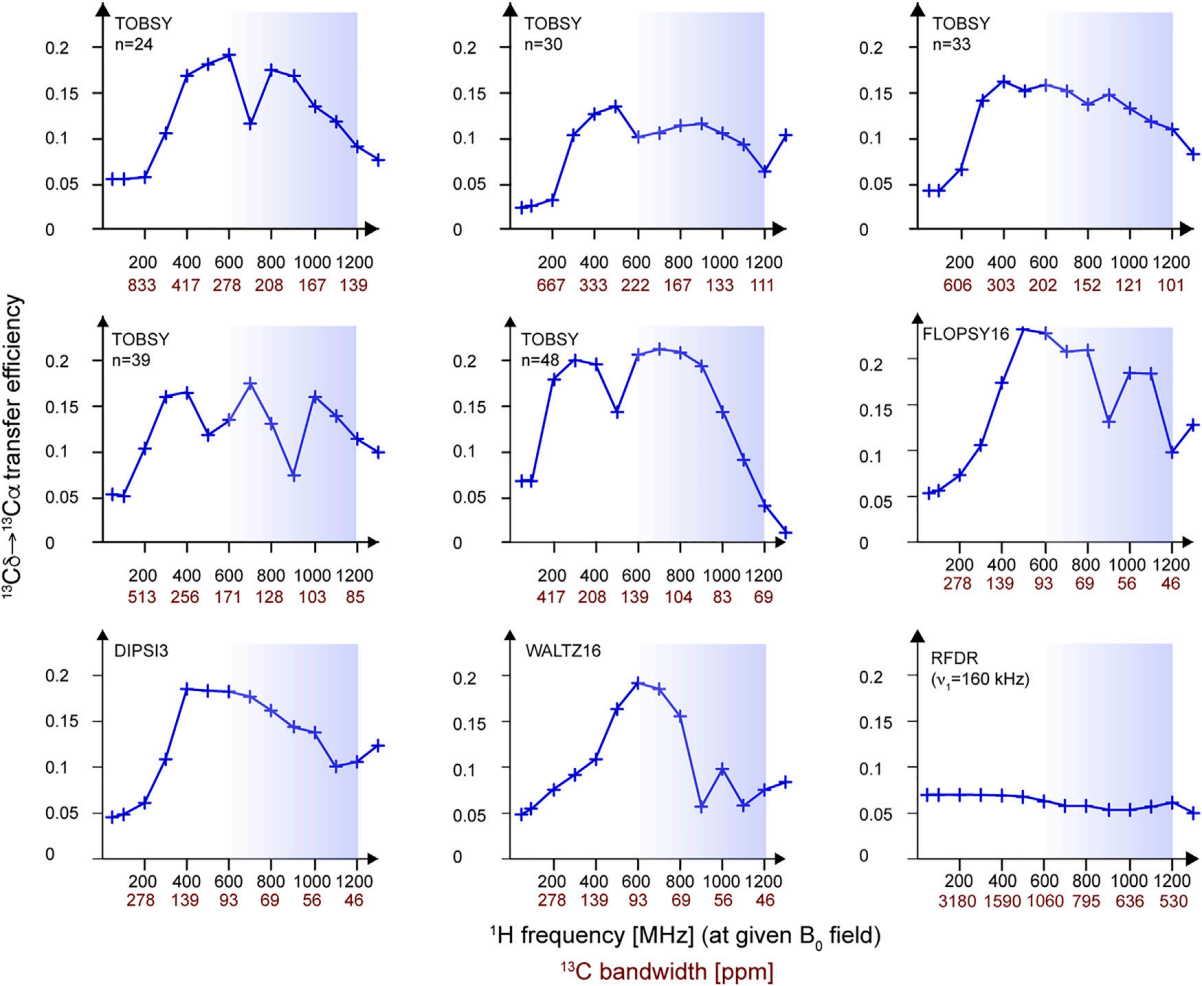
Efficiency of Cδ_1_→Cα transfer in a ^13^C_6_ spin system simulated in SIMPSON for a range of magnetic fields *B*_0_ (corresponding to ^1^H frequency between 50 and 1,300 MHz), MAS frequency of 55.5 kHz, and a constant RF strength of ν_1_ = ¼ ν_R_ ≈ 13.9 kHz. Note that, in essence, the dependence on ^13^C bandwidth (ν_1_ expressed in ^13^C ppm) of a particular mixing is investigated here. For each *B*_0_ point, a full buildup was performed, and an optimum was picked at the mixing times that are reported in Supplementary Figure S9. All RF schemes except the particularly computationally demanding DREAM were evaluated. Blue boxes indicate the *B*_0_ field range available for ^1^H-detected MAS NMR at present.

Despite apparent limitations, SIMPSON simulations still provide upper limits for transfer efficiencies, which is useful to predict performance trends at different experimental conditions. For example, ν_R_ dependence sampled between 30 and 200 kHz (Supplementary Figure S8) shows an optimal MAS range of 80–100 kHz for TOBSY and TOCSY schemes. The penalty observed at ν_R_ > 100 kHz is surprising, and in fact related to RF strength (ν_1_), which was fixed in a constant proportion to ν_R_. To isolate ν_1_ dependence without traversing through recoupling conditions, we performed additional simulations at *constant* ν_1_ = 55.5 kHz and ν_1_ = ¼ ν_R_, but indirectly varied RF bandwidth of sequences *via* modulation of resonance frequencies and CSA with the strength of *B*_0_ field (Figure 7). At high field (above ν_0,H_ = 800–1,000 MHz), most sequences show insufficient ^13^C bandwidth, thus decreasing the performance of a multibond transfer. At low field, the transfer efficiency degrades as well, with a very similar behavior to that observed in Supplementary Figure S8 for large rotation rates ν_R_. This suggests that the effect actually relates only to the effective ^13^C bandwidth, and we confirmed by observation of C′ magnetization that an excessive RF strength is detrimental to ^13^Cα magnetization due to the concurrent drainage to C′.

This leads to a paradox that increased MAS rates might not necessarily be beneficial if RF strength is bound to ν_R_ by the mixing design. Contrary to the case of TOBSY, TOCSY schemes tolerate variable ν_1_/ν_R_ ratios in a wide range below ¼ (Supplementary Figure S10), which gives an additional flexibility for their application at moderate-to-high fields and very fast spinning. The simulation does not account for RF-dependent ^13^C T_1ρ_, and very small RF should obviously also be avoided, particularly in systems showing increased microsecond local dynamics.

### 3.3 Methyl Resonance Assignment in Proteins by Correlation to Backbone Spins

Results obtained for fMLF strongly suggest proton dilution for the effective ^13^C mixing in extended spin systems in proteins. Fortunately, with fast MAS, this can be accomplished without compromising the occupancy of methyl ^1^H sites, e.g., by selective labeling of Ile, Leu, and Val residues from suitable precursors coupled to expression in D_2_O media (Tugarinov and Kay 2004). Importantly, ^13^C (and ^2^H)-enriched glucose must be used to preserve the continuity of Ile and Leu ^13^C side chains (Supplementary Figure S1), since α and β of the former and α and carbonyl carbons of the latter residue are incorporated from the medium rather than from the precursor (Lundström et al., 2007).

To demonstrate the efficacy of methyl ^1^H assignment, we resorted to a model SH3 domain of α-spectrin in a microcrystalline state. Given the selective ^1^H labeling, a magnetization transfer *from* methyl *to* backbone amide ^1^H (and *not* a reverse one) is strongly preferred for the sensitivity reasons. In addition to 3-fold larger occupancy of methyl ^1^H, the initial polarization is enhanced by fast *T*_1_ relaxation of methyl protons, while NMR signal detection is performed on well-dispersed, generally narrower, and, thus, more sensitive amide ^1^H protons. Naturally, a protocol for complete reprotonation of labile amide ^1^H sites after expression in D_2_O is a prerequisite for this approach. While extensive deuteration is not a necessity for proteins as simple as SH3 at fast MAS (>50 kHz) and high field (here 18.8 T), it would certainly be required for resolution and sensitivity reasons in more challenging applications. We employed a slightly adapted literature RF irradiation scheme (shown already in Figure 2D), with various ^13^C mixings as in the previous cases.

#### 3.3.1 Joint Effect of the Rotation Frequency and RF Strength

**FIGURE 8:**
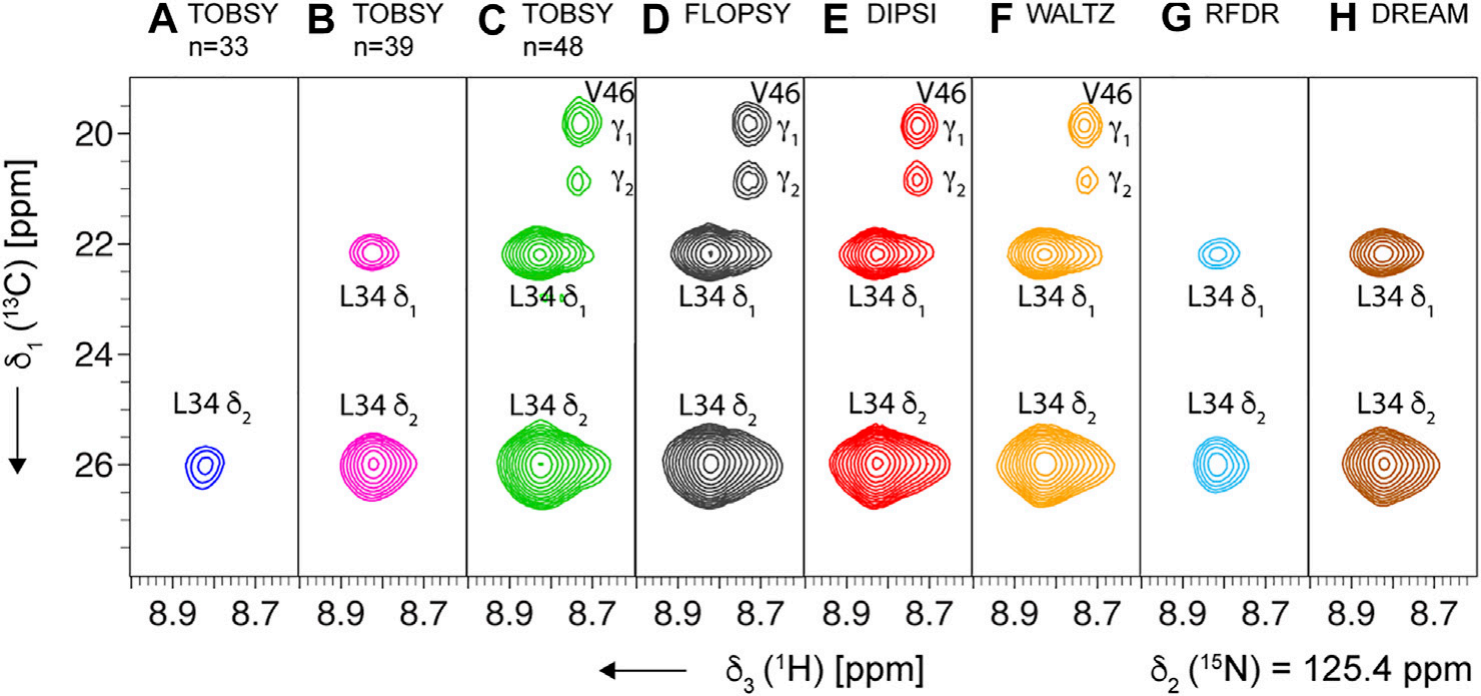
Representative strips from 3D (H)C(CC)(CA)NH experiment for SH3 protein recorded at MAS frequency of 94.5 kHz and on an 18.8-T spectrometer, using the following mixing schemes at their optimal mixing time (see Supplementary Table S3): **(A–C)** TOBSY C9^1^_*n*_, with *n* = 33, 39, and 48, respectively, **(D)** FLOPSY-16, **(E)** DIPSI-3, **(F)** WALTZ-16, **(G)** RFDR (ν_1_ = 100 kHz), and **(H)** DREAM. All planes show the cross-section at δ_2_ (^15^N) = 125.4 ppm, and show two cross-peaks for Leu-34 residue (L34 ^1^H^N^,^15^N intra-residue correlations to δ_1_ and δ_2_^13^C). The most intense spectra (panels **C–F**) additionally show cross-peaks of Val-46 residue due to partial overlap of L34 and V46 ^1^H^N^ and ^15^N frequencies.

For SH3 sample we acquired 3D spectra with site-specific resolution at a single optimized mixing time for each ^13^C mixing. Two spinning conditions, ν_R_ = 55.5 and 94.5 kHz, were applied using the same probe and rotor for the maximum cohesion of the data, and the representative strips are presented in Figure 8 (for ν_R_ = 94.5 kHz).

**FIGURE 9:**
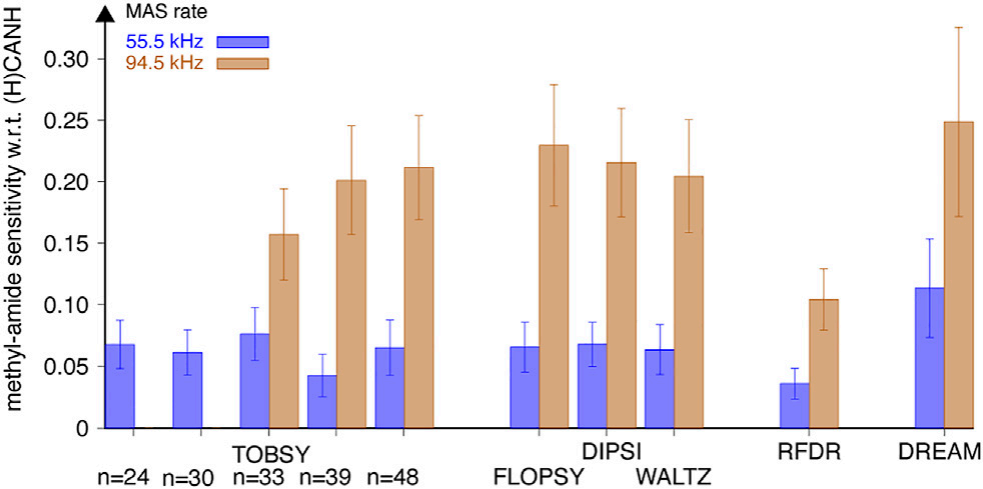
Sensitivity (S/N per unit time) of methyl-to-amide correlations in 3D (H)C(CC)(CA)NH spectra normalized to sensitivity of respective intraresidue correlations in 3D (H)CANH spectra, recorded for SH3 protein at a MAS frequency of 55.5 (*blue* bars, left in each pair) and 94.5 kHz (*tan* bars, right in each pair) on an 18.8-T spectrometer using various ^13^C mixing schemes. Relative intensity of 24 cross-peaks [for 6 valine (all except Val-46), 1 isoleucine, and 6 leucine residues] was averaged with equal weights. Data for TOBSY C9^1^_24_ and C9^1^_30_ were not collected at ν_R_ = 94.5 kHz. The error bars reflect the standard deviation (scatter) of values observed for a set of residues, not the experimental error of average sensitivity.

The experiment inherently shows only the transferred signal, thus poses difficulties to the rigorous quantification of ^13^C transfer efficiency. Nevertheless, we attempted to correct for uneven efficiency of the remaining part of the pulse sequence (notably CP steps) at different MAS conditions by normalizing the cross-peak intensities to those observed in the 3D (H)CANH experiment, which shares majority of the coherence pathway. The observed relative signal-to-noise ratio was averaged over 24 individual strong and resolved correlations. Results shown in Figure 9 (for all residue types) and Supplementary Figure S11 (separately for Val, Leu, and Ile) show a dramatic increase of ^13^C mixing efficiency with fast MAS (94.5 w. r. t. 55.5 kHz), with a similar effect (a factor of 3.2–3.5 for TOCSY and C9^1^_48_) to that observed on nondeuterated fMLF. Despite a dilute network of ^1^H interactions in the SH3 sample, and averaging also over valine and isoleucine residues, the best-performing RF schemes are virtually the same as for the leucine residue in fMLF (Figure 6), namely, DREAM at 55.5 kHz MAS, and DREAM, TOBSY C9^1^_48_, and three TOCSY sequences at 94.5 kHz MAS. The relevance of ^1^H–^1^H and ^1^H–^13^C interactions might be due to the fact that, despite partial sample deuteration, the local ^1^H environment of leucine, valine, and isoleucine residues is quite dense, with only 4 out of 11, 2 of 9, and 7 of 11 protons replaced by deuterons, respectively. As for the overall proton density, 14 ILV residues (out of 62 residues in SH3) contain 81 protons at methyl sites. These, in addition to 116 backbone and side-chain labile protons, yield a moderate rather than a low protonation level (approximately 38%).

Compared to fMLF, spins in SH3 protein undergo higher-amplitude dynamics, and certainly there is a larger contribution of incoherent effects, such as leading to ^13^C *T*_2_ and *T*_1ρ_ relaxation and ^1^H spin-diffusion. *T*_1ρ_ relaxation is further suppressed by stronger RF at increased MAS frequency (particularly for DREAM, which effectively spin-locks the ^13^C coherence), as it is bound to ν_R_ for all sequences except RFDR. As for the coherent effects, increased MAS frequency also more effectively averages *D*_CH_ interactions (i.e., a fast rotation suppresses second-order AHT cross-terms, deleterious, e.g., for TOBSY with low *n*). Definitely, there is an additional S/N benefit from line narrowing of amide ^1^H resonances at 94.5 kHz MAS (separable under a few of assumptions), but, as described below, it is estimated to contribute no more than 20% to the overall S/N gain.

In view of the apparent relevance of H–C and H–H interactions in deuterated SH3, additional efficiency gains are expected with the use of high-power ^1^H decoupling (ν_1,H_ > 3ν_R_) during ^13^C mixing; however, this likely endangers sample hydration and thus spectral quality. Overall, fast MAS seems to be an attractive route to amplify the signal, and, in our comparison, the gain in efficiency well compensates the 2- to 3-fold sensitivity losses entailed by active volume reduction from a 1.3-mm rotor (optimal for ν_R_ ≈ 55 kHz) to a 0.7- or 0.81-mm rotor (ν_R_ ≈ 100 kHz). We speculate that for methyl resonance assignment using the presented approach, *non*deuterated proteins would benefit from fast MAS to an even larger extent.

#### 3.3.2 Effect of the Static Magnetic Field

**FIGURE 10:**
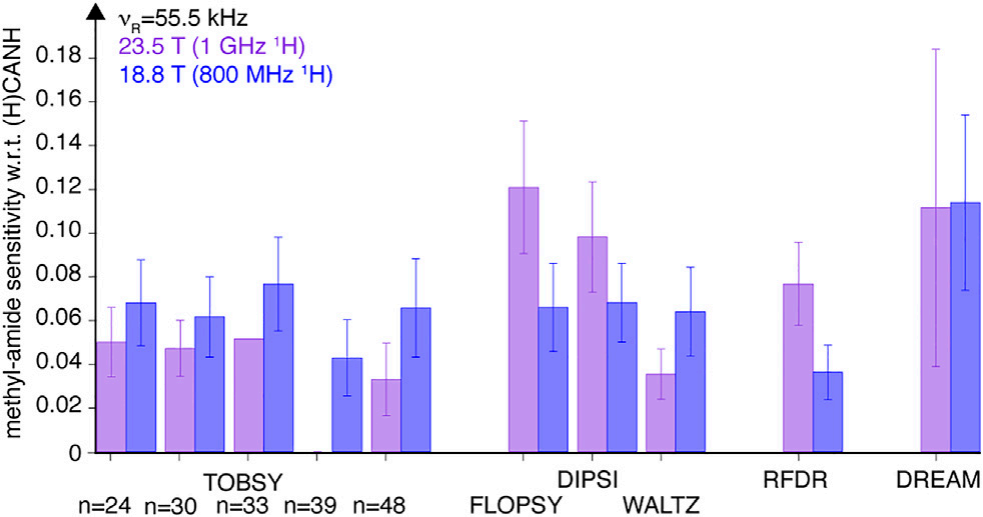
Efficiency of various ^13^C mixing schemes compared between 18.8- and 23.5-T fields [*purple* (left) and *blue* (right) bars, respectively]. Presented are sensitivity of correlation peaks in 3D (H)C(CC)(CA)NH spectra of SH3 protein obtained at 55.5 kHz MAS, with respect to sensitivity of correlations in (H)CANH experiment at both conditions. Relative sensitivity is averaged equally over 24 cross-peaks of Val, Leu, and Ile residues. Data for TOBSY C9^1^_39_ were not collected at 23.5 T field. The error bars reflect the standard deviation (scatter) of values observed for a set of residues, not the experimental error of average sensitivity.

Our experiments were performed at a typical high-field spectrometer (18.8 T) used commonly in protein studies by ^1^H-detected MAS. Stronger *B*_0_ fields are increasingly accessible; therefore, it is relevant to explore the performance of ^13^C mixing at such conditions. For example, for a transition to a 23.5-T field, spin dynamics simulations on a six-^13^C spin system suggest only a minor and negative impact on transfer efficiency (results in Figure 7 are summarized for two considered *B*_0_ fields in Supplementary Figure S12). For experimental verification, we performed a full series of 3D experiments on SH3 protein (from the same crystallization batch) in a 1.3-mm rotor spun at ν_R_ = 55.5 kHz on a 1,000-MHz ^1^H spectrometer. Data were analyzed with a site-specific resolution and averaged over all reliable cross-peaks of Leu, Val, and Ile residues as mentioned above. Also, as before, we normalized the obtained S/N ratios of cross-peaks with respect to peaks in 3D (H)CANH to eliminate the effects of larger Boltzmann polarization, rotor active volume, possibly different sample packing density, and specific RF coil efficiency (see Supplementary Material). Results compared in Figure 10 illustrate that only selected ^13^C mixing schemes, notably, FLOPSY, RFDR, and DIPSI, actually profit from higher *B*_0_ field. Note that for consistency with the data acquired at 18.8 T, we did not optimize RF strength (ν_1_) for TOCSY sequences (in the range below ¼ ν_R_), despite potential gains in efficiency (see Figure 7 and Supplementary Figure S10). The rationale for observed efficiency changes are again coherent and incoherent effects of H–H and H–C interactions, which evade proper treatment in our spin dynamics simulations.

#### 3.3.3 Linearization of the ^13^C Side Chain

One of the reasons for a low transfer efficiency between methyl and alpha ^13^C spins using *J*_CC_-based isotropic mixing in Leu, Val, and Ile is a branched topology of the ^13^C chain. A 2-fold degradation of FLOPSY-8 efficiency for Ile residues was identified by Kay and co-workers, and remedied with relay-type (COSY) experiments in which ^13^C coherence is sequentially transferred with a careful manipulation of individual ^13^C spins using very frequency selective pulses (Tugarinov and Kay 2003b). This approach is not immediately applicable in MAS NMR, since required transfer delays are prohibitively long compared to ^13^C *T*_2_ lifetimes. For Leu residues, the overlap between chemical shifts of δ and γ ^13^C precludes the elimination of detrimental passive coupling to the second methyl ^13^C; thus, in solution NMR, the issue was addressed by a tailored amino acid labeling, in which one of the methyl groups was labeled as ^12^CD_3_ (Tugarinov and Kay 2003a). Since the labeling of the α-ketoisovalerate (precursor) is not stereospecific, a 1:1 mixture of Leu (^13^CH_3_, ^12^CD_3_) and Leu (^12^CD_3_,^13^CH_3_) isotopomers is obtained (and analogously for valines), entailing a 50% loss of methyl ^1^H occupancy. Nevertheless, this sensitivity loss was compensated by larger efficiency of complex out-and-back transfer schemes proposed by Kay and co-workers for methyl ^1^H assignment in solution.

To verify the utility of the linearization of ^13^C side chains of Leu and Val residues for MAS NMR, we first performed SIMPSON simulations, and compared the behavior of 5-^13^C spin (linear) and 6-^13^C spin (branched) spin systems that mimic the leucine residue with respective isotope labeling patterns (Supplementary Figures S13 and Supplementary Figure S14). Indeed, for all *J*-based isotropic mixing schemes (TOBSY and TOCSY), a 2- to 3-fold efficiency gain is predicted at ν_R_ = 100 kHz, and comparable at ν_R_ = 55.5 kHz. It is thus expected that, in this case, the 50% loss of initial signal is at least compensated by increased Cδ→Cα (Cγ→Cα for valines) transfer efficiency. Dipolar-based mixing (RFDR and DREAM) marginally profits from the simplification of ^13^C chain, likely only due to dispersion of ^13^C magnetization over a smaller number of ^13^C spins.

**FIGURE 11:**
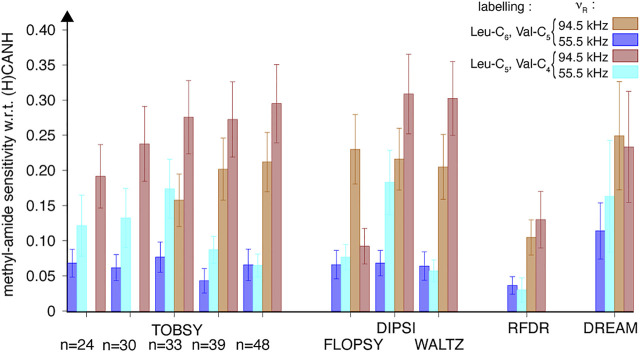
Relative efficiency of ^13^C mixing schemes compared between two SH3 protein samples: with branched (“ILV-C5”, *tan* filled bars) and linear (“ILV-C4”, *red* filled bars) ^13^C spin systems of leucine and valine residues, quantified using 24 cross-peak intensities in 3D (H)C(CC)(CA)NH spectra recorded at a MAS frequency of 94.5 kHz on an 18.8-T spectrometer. Data for 55.5 kHz MAS is shown as *blue* and *cyan* bars for “ILV-C5” and “ILV-C4” SH3 samples, respectively. Sensitivity of each cross-peak was normalized to respective intra-residue peak in 3D (H)CANH spectrum at each sample and spinning condition, and subsequently averaged over Val, Leu, and Ile residues.

The sample of SH3 with linearized side chains of Leu and Val [formally {I (δ_1_), L (^13^CH_3_, ^12^CD_3_), V (^13^CH_3_,^12^CD_3_)} U-(^15^N, ^13^C, ^2^H, ^1^H^N^)-labeled, with Ile-^13^C_6_, Leu-^13^C_5_,Val-^13^C_4_, here referred to as “ILV-C4” for brevity] was prepared by following carefully the expression protocol of the previous (“ILV-C5”, in fact Ile-^13^C_6_, Leu-^13^C_6_, and Val-^13^C_5_) sample, but using the 1,2,3,4-^13^C-3,4′,4′,4′-^2^H-labeled α-ketoisovalerate as a precursor (Tugarinov and Kay 2003a). The microcrystals were paramagnetically doped with EDTA-chelated Cu^II^ ions, and transferred into MAS 0.81-mm rotor with comparable density (see ^13^C 1D spectra of both samples in Supplementary Figure S15A). As expected, the methyl ^1^H signal in direct-excitation spectra decreased approximately twice (Supplementary Figure S15B). We repeated the entire series of 3D (H)C(CC)(CA)NH experiments at both spinning frequencies ν_R_ = 55.5 and 94.5 kHz, using virtually the same previous experimental settings. Differences in CP efficiency and rotor packing density were compensated by normalization to the respective intraresidue peak intensities in (H)CANH spectra. Finally, for each condition, the relative cross-peak intensities that approximate Cδ (or Cγ)→Cα transfer efficiencies were averaged over 24 correlation peaks. The comparison in Figure 11 provides evidence that transfer efficiency increased well beyond the factor of 2, yielding improved sensitivity for “ILV-C4” sample (with the exception of FLOPSY), and unexpectedly also manifested for dipolar-based mixings, for which a total sensitivity loss was expected based on SIMPSON simulations. Despite the 50% dilution of methyl ^1^H spins, “ILV-C4”-labeling clearly yields superior results, with DIPSI, WALTZ, and TOBSY C9^1^_48_ as the methods of first choice. Interestingly, if the stereospecifically labeled ^13^C_4_-α-ketoisovalerate was commercially available, S/N ratios could double, corresponding to a further sensitivity gain (i.e., per square root of time) of √2 (accounting for the need for two separate acquisition series).

**FIGURE 12:**
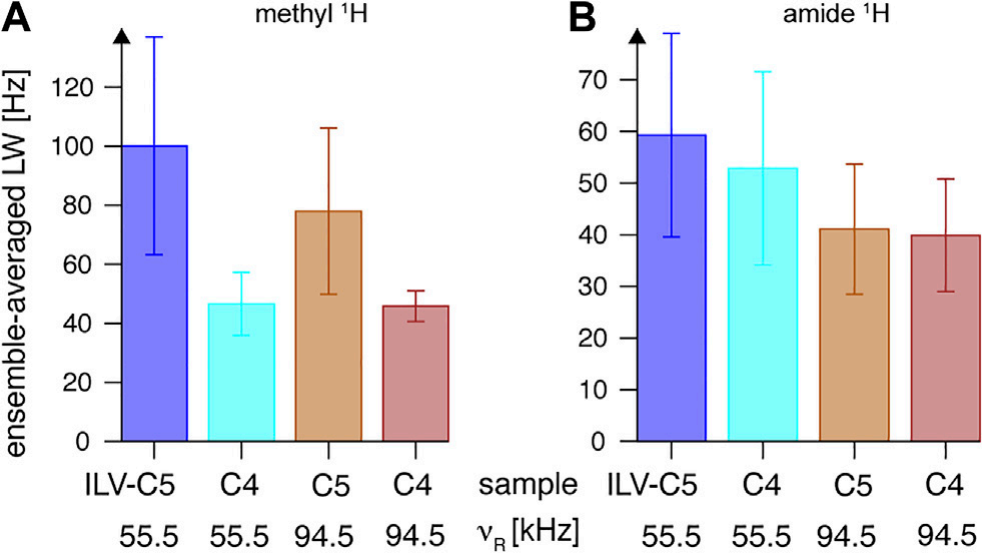
Linewidths (full widths at half height) of **(A)** methyl and **(B)** backbone amide ^1^H resonances observed in 2D ^13^C-^1^H and ^15^N–^1^H CP-HSQC spectra, respectively, for “ILV-C4” (at ν_R_ = 55.5 kHz in *cyan*, and at ν_R_ = 94.5 kHz in *red*) and “ILV-C5” (at ν_R_ = 55.5 kHz in *blue,* and at ν_R_ = 94.5 kHz in *tan*) SH3 samples on an 18.8-T spectrometer. Linewidths observed in each case were averaged over all resonances, and the error bars indicate the standard deviation (the dispersion of values observed for all residues), not the precision of linewidth determination.

The unexpectedly high gains observed with “ILV-C4” labeling can be explained by (1) decreased proton density, particularly in the local environments of leucine and valine ^13^C spin systems, and (2) increased detection sensitivity due to amide ^1^H line narrowing. The relevance of the first effect on the efficiency of ^13^C transfer can be appreciated based on the MAS frequency dependence discussed above for the “ILV-C5” sample. Here, we illustrated the effect by measurement of methyl ^1^H linewidth change upon additional proton dilution, as observed in ^1^H, ^13^C-CP-HSQC spectra as cross-peak raw full-width at half-height. Indeed, a 2-fold linewidth reduction is observed at ν_R_ = 55.5 kHz (Figure 12A and Supplementary Figure S16A, B), surpassing the effect of faster rotation for the “ILV-C5” sample (the ratio of 1.3 between 55.5 and 94.5 kHz). For the “ILV-C4” sample, both spinning conditions lead to similar linewidths (46 ± 11 Hz) with only few exceptions.

The second effect was carefully quantified by ^1^H amide linewidth measurements in fingerprint ^1^H, ^15^N-CP-HSQC spectra (also without application of a window function in ^1^H dimension). As expected, proton dilution in the methyl side chain of Leu and Val residues has a relatively smaller impact on amide ^1^H coherence lifetimes (Figure 12B). At ν_R_ = 94.5 kHz, both samples show similar linewidths of 40 Hz (see Supplementary Figures S16C,D for a per-peak comparison), but approximately 9% difference is observed at ν_R_ = 55.5 kHz. The line-narrowing effect of rotation frequency increase is of a factor of 1.4 and 1.3 for “ILV-C5” and “ILV-C4” samples, respectively. Overall, the use of “ILV-C4” labeling is beneficial for resolution of ^1^H, ^13^C methyl resonances, and sensitivity of methyl-to-amide correlations.

#### 3.3.4 Assignment of Protons: 4D Correlations

**FIGURE 13:**
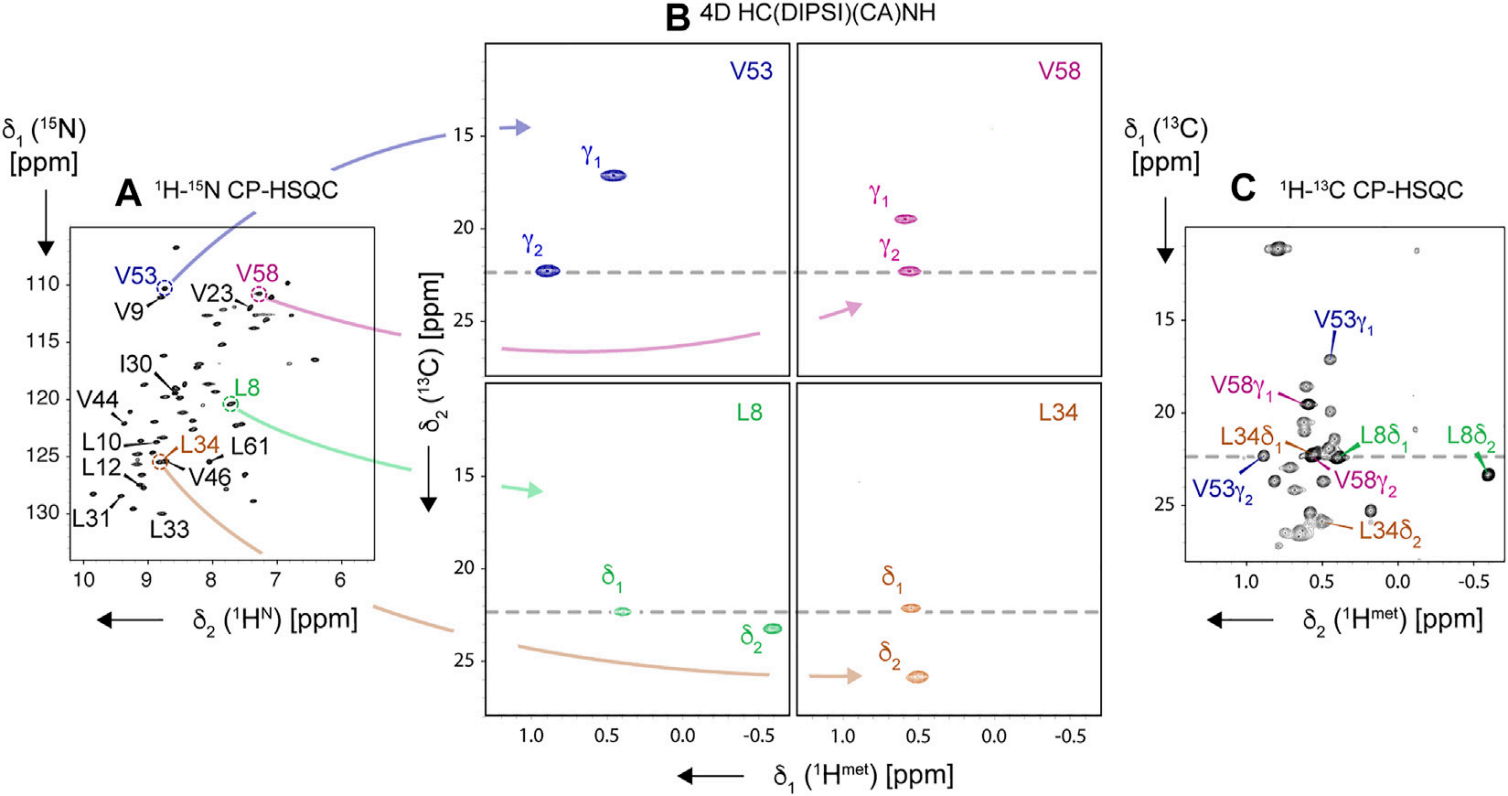
Resolving ambiguity of methyl ^1^H and ^13^C resonance assignment using 4D HC(DIPSI)(CA)NH experiment, demonstrated on the “ILV-C4” sample of SH3 protein, spun at 94.5 kHz on an 18.8-T spectrometer. Navigation in the 4D spectral matrix is based on peak positions in 2D ^1^H, ^15^N-CP-HSQC **(A)**, allowing to display intraresidue pairs of correlations in ^1^H, ^13^C-cross sections of the 4D cube **(B)**. Obtained methyl resonance assignments for four representative residues (L8, L34, V53, and V58, color-coded) are readily transferable to 2D ^1^H, ^13^C-CP-HSQC spectrum **(C)**. In **(A)**, amide correlations of all Leu, Val, and Ile residues are labeled. All correlations except for residue Val-46 were observed in the 4D experiment.

As shown in Figure 13, even in proteins as small as SH3, a single 3D (^13^C^met^-edited) spectrum can result in massive ambiguities. For example, V53 γ_2_, V58 γ_2_, L8 δ_1_, and L34 δ_1_^1^H assignment cannot be established from (H)C(CC)(CA)NH spectrum alone, since these methyl sites exhibit close ^13^C shifts (highlighted by a *dashed* line in ^13^C-CP-HSQC, panel C). The joint analysis of a pair of 3D (H)C(CC)(CA)NH and H(C)(CC)(CA)NH spectra could help to correctly match ^1^H to ^13^C frequencies within a spin system (given sufficient resolution of each spectrum); however, if both methyl sites in a residue show either close ^1^H or close ^13^C shifts, the ambiguity remains (e.g., in Val-58). The stereospecific labeling, i.e., using leucine and valine pro-*S*-C_4_ and pro-*R*-C_4_ precursors (yet unavailable for the presented approach) would be a costly solution.

Alternatively, one can resort to high-resolution 4D spectroscopy with non-uniform sampling, increasingly popular for MAS NMR (Huber et al., 2011; Linser et al., 2011; Paramasivam et al., 2012; Linser et al., 2014; Xiang et al., 2014; Fraga et al., 2017; Sergeyev et al., 2017; Marchanka et al., 2018; Vasa et al., 2018). Indeed, the use of a 4D HC(CC)(CA)NH spectrum (Figure 13B) allows disambiguating the assignment as demonstrated for L34 and V58 spin systems (close ^1^H shifts), which additionally suffer from overlap of L34 δ_1_ and V58 γ_2_ peaks in ^1^H, ^13^C-CP-HSQC. Given that a 4D NUS spectrum can be acquired in a similar time to a pair of 3D ones, this approach is recommended for all but the smallest proteins.

In the presented 4D NUS experiment, the average S/N of cross-peaks, normalized to 24 h of acquisition, was 25 ± 7 (Supplementary Figure S17). One can safely interpolate that a minimal S/N ratio of 10 is obtained in 3.8 h for SH3 (62 aa). If we limit the discussion to the class of microcrystalline samples of comparable spectral properties (CP efficiency and linewidths) and crystal packing density, S/N of a single peak scales inversely with the number of amino acids *K* (to account for the smaller number of molecules in a rotor). Thus, the time needed for the same minimal S/N ratio scales as *K* ^2^, yielding reasonable acquisition times of 0.42, 1.7, 3.7, 6.7, 10.4, and 15 days needed at 18.8 T for proteins of *K* = 100, 200, 300, 400, 500, and 600 residues, respectively. It is noteworthy that these times would be approximately 2- and 3.4-fold shorter, respectively, on 23.5- and 28.2-T (1,000 and 1,200 MHz ^1^H) spectrometers available nowadays (assuming *B*_0_^3/2^ scaling of S/N ratio). Our results thus suggest feasibility of site-specific assignment of methyl resonances in sizable proteins for which the crystallization conditions and deuteration protocol have been already established, and can potentially be of great value to further solution or solid-state NMR studies. Whether methyl chemical shifts are readily transferable to other sample conditions is yet to be investigated. However, provided no significant alteration to protein fold, aliphatic ^13^C should not experience large deviations as demonstrated recently for maltose binding protein (Stanek et al., 2020).

## 4 Conclusion

We showed that a careful selection of ^13^C homonuclear mixing allows one to obtain sensitive correlations of methyl-to-amide ^1^H chemical shifts under fast MAS and high *B*_0_ field conditions. For highly deuterated proteins on an 18.8-T spectrometer, the best performance was obtained with DIPSI-3, WALTZ-16, and TOBSY C9^1^_48_ at ν_R_ = 94.5 kHz, and with DREAM at ν_R_ = 55.5 kHz. Dramatic improvement of the multi-bond (methyl to alpha ^13^C) transfer efficiency was observed upon increase of MAS frequency from 55.5 to 94.5 kHz, which is attributed to the suppression of incoherent effects of H–H and H–C dipolar interactions. Further significant performance increase was obtained by the linearization of ^13^C side chains of leucine and valine residues, with additional gains in resolution of methyl ^1^H resonances. We demonstrated that unambiguous assignment of methyl ^1^H and ^13^C resonances is feasible for microcrystalline proteins by a combination of protein deuteration, paramagnetic *T*_1_ relaxation enhancement, suitable ^13^C isotope labeling, ultra-fast MAS, and 4D spectroscopy with non-uniform sampling. These findings pave the way for efficient assignment complementary to the labor-intensive mutagenesis-based strategy, with protein mass limitations largely mitigated by the favorable scaling of sensitivity in MAS NMR.

## Data Availability

The datasets presented in this study can be found in online repositories. The names of the repository/repositories and accession number(s) can be found at: https://doi.org/10.5281/zenodo.5911897.
