# Supplementary material for: NMR Assignment of Methyl Groups in Immobilized Proteins Using Multiple-Bond 13C Homonuclear Transfers, Proton Detection, and Very Fast MAS
Source: Front Mol Biosci. 2022 Mar 29;9:828785. doi: 10.3389/fmolb.2022.828785 (PMC9002630; doi:10.3389/fmolb.2022.828785)
Supplement: Supplementary file 1 [file DataSheet1.PDF]

Supplementary Material

1 Supplementary Data

1.1 NMR data acquisition and Fourier processing parameters

Supplementary Table 1. Experimental parameters for ^{13}C-detected 2D experiments.

General acquisition parameters				
Sample	-	2,3-^{13}C-Ala	U-^{13}C,^{15}N-fMLF	
Field	[T]	14.1	18.8	18.8
Probe	-	Bruker H/C/N/D 1.3 mm	Darklands OU H/C/N/D 0.81 mm	
NS	-	8		
Spinning speed	[kHz]	55.555	55.5	94.5
Relaxation delay	[s]	1.5	1.9	
TD F2 (13C)	[point]	2048		
SW F2 (13C)	[ppm] [kHz]	92.0 13.889	207.2 41.667	207.2 41.667
AQ F2 (13C)	[ms]	73.8	24.63	
O2P (13C)	[ppm]	37.9	35.0	
TD F1 (13C)	[point]	64	128	
SW F1 (13C)	[ppm]	46.0	64.58	
AQ F1 (13C)	[ms]	4.6	4.93	
^{1}H 90° p.w.	[µs]	1.49	1.29	1.26
^{13}C 90° p.w.	[µs]	3.00	3.10	3.66
^{1}H RF during CP	[kHz]	142.8	148.0 (≈5/2vR)	178.3 (≈7/4vR)
^{13}C RF during CP	[kHz]	93.5	80.7 (≈3/2vR)	68.3 (≈3/4vR)
^{1}H shape during CP	-	Ramp from 90 to 100%		

Supplementary Table 2. NMR data processing parameters for ^{13}C-detected 2D experiments.

		Data set		
Sample	-	2,3-^{13}C-Ala	U-^{13}C,^{15}N-fMLF	
Spinning speed	[kHz]	55.555	55.5	94.5
SI F2 (^{13}C)	[point]	2048	4096	4096
Digital resolution F2 (^{13}C)	[Hz/point]	6.8	20.2	
SI F1 (^{13}C)	[point]	256	1024	
Digital resolution F1 (^{13}C)	[Hz/point]	27.1	12.7	
Lorentzian broadening in F2 (^{13}C)	[Hz]	33	50	
Apodization function in F1 (^{13}C)	-	Squared sine bell	Squared sine bell	
Shift of apodization function F1 (^{13}C)	[°]	90	90	

Supplementary Table 3. Experimental parameters for 3D ^{1}H-detected SH3 protein experiments.

			Data set				
Sample labelling	-	-	**U-^{13}C,^{15}N,^{2}H, ^{1}H^N, I, L-^{13}C6, V-^{13}C5**		**U-^{13}C,^{15}N,^{2}H, ^{1}H^N, I-^{13}C6, L-^{13}C5, V-^{13}C4**		**U-^{13}C,^{15}N,^{2}H, ^{1}H^N, I, L-^{13}C6, V-^{13}C5**
Field	[T]	-	18.8				23.5
Probe	-	-	Darklands OU H/C/N/D 0.81 mm				Bruker H/C/N/D 1.3mm
Spinning speed	[kHz]	-	55.5	94.5	55.5	94.5	55.555
**General acquisition parameters**							
Parameter	Unit	Parameter optimized?					
NS	-	-	8 or 16^a	8	8	16	16
relaxation delay	[s]	-	0.3	0.4	0.3	0.4	0.3
TD F3 (^{1}H)^b	[-]	NO	2048				4096
SW F3 (^{1}H)	[ppm]<br>[kHz]	NO	31.26<br>25.0				99.98<br>100.0
AQ F3 (^{1}H)	[ms]	NO	40.96				20.53
O1P (^{1}H)	[ppm]	NO	4.716	4.716	4.654	4.716	4.77
TD F2 (^{13}C)	[-]	NO	64				80
SW F2 (^{13}C)	[ppm]	NO	20.053				20.0
AQ F2 (^{13}C)	[ms]	NO	7.936				7.95
O2P (^{13}C)	[ppm]	NO	18.975				16
TD F1 (^{15}N)	[-]	NO	128				128
SW F1 (^{15}N)	[ppm]	NO	32.051				30.0
AQ F1 (^{15}N)	[ms]	NO	24.64				21.0
O3P (^{15}N)	[ppm]	NO	120				118.5
^{1}H 90° pw	[µs]	YES	2.547	2.461	2.655	2.674	2.470
^{13}C 90° pw	[µs]	YES	2.412	2.467	2.397	2.469	4.920

^{15}N 90° pw	[μs]	YES	7.050	7.206	7.190	6.815	6.250
**Cross-polarization**							
1	$^{1}H \rightarrow ^{13}C$ CP						
^{1}H RF strength (max)	[kHz]	YES	74.7 (≈5/4v_R)	128.3 (≈5/4v_R)	74.2 (≈5/4v_R)	123.7 (≈5/4v_R)	99.6 (≈5/2v_R)
^{1}H RF modulation	-	NO	Ramp from 90 to 100%				
^{13}C RF strength	[kHz]	NO	14.2 (≈1/4v_R)	24.1 (≈1/4v_R)	14.4 (≈1/4v_R)	24.1 (≈1/4v_R)	37.6 (≈2/3v_R)
contact time	[ms]	YES	7	4.75	5.0	5.0	3.0
2	$^{13}C \rightarrow ^{15}N$ CP						
^{13}C RF strength	[kHz]	NO	37.8 (≈2/3 v_R)	64.4 (≈2/3 v_R)	38.3 (≈2/3 v_R)	64.3 (≈2/3 v_R)	13.9 (≈1/4 v_R)
^{15}N RF strength (max)	[kHz]	YES	21.6 (≈1/3 v_R)	35.6 (≈1/3 v_R)	21.5 (≈1/3 v_R)	35.9 (≈1/3 v_R)	44.7 (≈3/4v_R)
^{15}N RF modulation	-	NO	Ramp from 70 to 100%				
contact time	[ms]	YES	20.0	20.0	20.0	20.0	13.0
3	$^{15}N \rightarrow ^{1}H$ CP						
^{1}H RF strength (max)	[kHz]	YES	72.2 (≈5/4v_R)	128.3 (≈5/4v_R)	74.2 (≈5/4v_R)	126.6 (≈5/4v_R)	89.8 (≈5/3v_R)
^{1}H RF modulation	-	NO	Ramp from 90 to 100%				
^{15}N RF strength	[kHz]	NO	14.4 (≈1/4v_R)	21.4 (≈1/4v_R)	14.1 (≈1/4v_R)	22.6 (≈1/4v_R)	37.0 (≈2/3v_R)
Contact time	[ms]	YES	0.5	0.5	0.5	0.5	0.5
**^{13}C mixing time**							

TOBSY n=24	[ms]	YES	19.01	16.25	15.55	19.30	12.1
TOBSY n=30	[ms]	YES	18.36	not measured	16.20	18.4	13.5
TOBSY n=33	[ms]	YES	16.53	16.76	19.01	20.95	not measured
TOBSY n=39	[ms]	YES	16.58	16.51	18.25	18.98	not measured
TOBSY n=48	[ms]	YES	17.28	16.25	19.01	19.30	12.1
DIPSI-3	[ms]	YES	19.56	18.40	19.56	20.70	19.6
FLOPSY-16	[ms]	YES	16.69	17.95	20.35	15.95	20.3
WALTZ-16	[ms]	YES	20.74	19.30	17.28	21.33	13.8
fpRFDR	[ms]	YES	8.64	5.42	5.18	5.76	9.2
DREAM	[ms]<br>[offset]	YES<br>YES	25.0 (18ppm)	24.5 (18ppm)	22.0 (22ppm)	25.0 (20ppm)	22.5 (28ppm)

[a] NS=16 for all experiments in this series except DREAM and TOBSY $C9^1_{39}$ and $C9^1_{33}$ (NS=8). [b] For convenience, the 4D experiment was implemented with ^{1}H indirect dimension in F3 (despite being the first indirect evolution period).

Experimental parameters for 4D HC-DIPSI-(CA)NH experiment. The pulse powers and lengths, delays, the mixing time, and RF offsets were fully consistent to 3D (H)C-DIPSI-(CA)NH experiment (reported in Supplementary Table 3). 8 scans were coherently added giving a total experiment time of 40 h. Spectral widths were set to 31.3, 14.0, 20.0, and 32.0 ppm for F4 (^{1}H), F3 (^{1}H), F2 (^{13}C), and F1(^{15}N), respectively. The spectrum was acquired using non-uniformly sampling of indirect evolution space, with 4800 hypercomplex points (1% of full Nyquist grid points) selected from 140 x 96 x 71 grid using Gaussian distribution function (with σ=0.5) for reduced variable $x^i = t^i / t^i_{max}$. (i=1, 2, 3). Maximum evolution time in the indirect dimensions was equal to 12.5, 11.9, 27.3 ms for F3(^{1}H), F2(^{13}C), F1(^{15}N), respectively.

Supplementary Table 4. NMR data processing parameters for 3D ^{1}H-detected SH3 protein experiments.

		Data set				
Field	[T]	18.8				23.5
Spinning speed	[kHz]	55.5	94.5	55.5	94.5	55.555
Parameter	Unit					
SI F3 (^{1}H)	[-]	4096				4096
Digital resolution F3 (^{1}H)	[Hz/point]	6.1				24.4
SI F2 (^{13}C)	[point]	256				256
Digital resolution F3 (^{13}C)	[Hz/point]	15.8				19.6
SI F1 (^{15}N)	[point]	512				512
Digital resolution F1 (^{15}N)	[Hz/point]	5.1				5.9
Lorentzian broadening in F3 (^{1}H)	[Hz]	50				50
Apodization function in F2 (^{13}C)	-	Squared sine bell				Squared sine bell
Shift of apodization function F2 (^{13}C)	[°]	90				90
Apodization function in F1 (^{15}N)	-	Squared sine bell	Squared sine bell	Squared sine bell	Squared sine bell	Squared sine bell
Shift of apodization function F1 (^{15}N)	[°]	90	90	90	90	90

1.2 Representative SIMPSON simulation scripts

1.2.1 C_2H_2 spin system (FLOPSY simulation)

```
#FLOPSY build-up
#For 4 spin system containing 2x13C and 2x1H (L-Ala like spin system)
#With possibility of switching off/on interactions: J_CC, D_CC, J_HC, D_HC, D_HH
#Copyright Piotr Paluch CNBCh University of Warsaw 2020
##crystal file rep168, gamma angle 11 (checked for convergence)
spinsys {
channels 13C
nuclei   13C 13C 1H 1H
#CA
shift 1 56.8p -60.0p 0.89 0 0 0
#CB
shift 2 24.6p -13.3p 0.29 0 0 0
#HA
shift 3 4.5p -5p 0 0 0 0
#HB
shift 4 1.5 -3p 0 0 0 0
dipole 1 2 -2084.9 0 92.756 -93.655

dipole 1 3 -22000.0 0 60.0 90.0
dipole 1 4 -666.0 0 70.0 80.0

dipole 2 3 -2000.0 0 70.0 80.0
dipole 2 4 -7300.0 0 60.0 80.0

dipole 3 4 -666.0 0 80.0 65.0

jcoupling 1 2 33.0 0 0 0 0 0
jcoupling 1 3 145.0 0 0 0 0 0
jcoupling 2 4 145.0 0 0 0 0 0
}


par {
method dsyev
conjugate_fid false
   proton_frequency 600e6
```

```
    spin_rate           55555.55555
    np                          1
    crystal_file        rep168
    gamma_angles                11
    start_operator              I1z
    detect_operator             I2z
    verbose                     1101
    # Spin system size
    variable number_of_spin 2
    #####
    variable out_factor 1
    variable C_offset 0.0
    variable RF_Frac_RO    0.25
    ######
variable flag_no_CC_J 0
variable flag_no_HC_J 1
variable flag_no_HC_D 1
variable flag_no_HH_D 1
variable flag_no_CC_D 1
}

proc pulseq {} {
global par
maxdt 0.1

set CRF [expr $par(RF_Frac_RO)*$par(spin_rate)]
set C90  [expr 0.25e6/$CRF]
set flip_table {46.0 96.0 164.0 159.0 130.0 159.0 164.0 96.0 46.0 }
set phase1_table {0.0 45.0 67.5 315.0 22.5 315.0 67.5 45.0 0.0}
set phase2_table {0.0 180.0 180.0 0.0 0.0 0.0 180.0 180.0 180.0 0.0 0.0 180.0 180.0 180.0
0.0 0.0}

reset
store 2

for {set m 1} {$m<=[expr $par(np)/$par(out_factor)]} {incr m} {
reset
prop 2
```

```
if {$par(flag_no_CC_J)==1} {
turnoff jcoupling_1_2
}

if {$par(flag_no_HC_J)==1} {
turnoff jcoupling_1_3
turnoff jcoupling_2_4
}

if {$par(flag_no_HC_D)==1} {
turnoff dipole_1_3
turnoff dipole_2_4
turnoff dipole_1_4
turnoff dipole_2_3
}

if {$par(flag_no_HH_D)==1} {
turnoff dipole_3_4
}

if {$par(flag_no_CC_D)==1} {
turnoff dipole_1_2
}

offset $par(C_offset)
#0
pulse [expr 46.0*$C90/90.0] $CRF 0.0
pulse [expr 96.0*$C90/90.0] $CRF 45.0
pulse [expr 164.0*$C90/90.0] $CRF 67.5
pulse [expr 159.0*$C90/90.0] $CRF 315.0
pulse [expr 130.0*$C90/90.0] $CRF 22.5
pulse [expr 159.0*$C90/90.0] $CRF 315.0
pulse [expr 164.0*$C90/90.0] $CRF 67.5
pulse [expr 96.0*$C90/90.0] $CRF 45.0
pulse [expr 46.0*$C90/90.0] $CRF 0.0
#180
pulse [expr 46.0*$C90/90.0] $CRF 180.0
```

```
pulse [expr 96.0*$C90/90.0] $CRF 225.0
pulse [expr 164.0*$C90/90.0] $CRF 247.5
pulse [expr 159.0*$C90/90.0] $CRF 495.0
pulse [expr 130.0*$C90/90.0] $CRF 202.5
pulse [expr 159.0*$C90/90.0] $CRF 495.0
pulse [expr 164.0*$C90/90.0] $CRF 247.5
pulse [expr 96.0*$C90/90.0] $CRF 225.0
pulse [expr 46.0*$C90/90.0] $CRF 180.0
#180
pulse [expr 46.0*$C90/90.0] $CRF 180.0
pulse [expr 96.0*$C90/90.0] $CRF 225.0
pulse [expr 164.0*$C90/90.0] $CRF 247.5
pulse [expr 159.0*$C90/90.0] $CRF 495.0
pulse [expr 130.0*$C90/90.0] $CRF 202.5
pulse [expr 159.0*$C90/90.0] $CRF 495.0
pulse [expr 164.0*$C90/90.0] $CRF 247.5
pulse [expr 96.0*$C90/90.0] $CRF 225.0
pulse [expr 46.0*$C90/90.0] $CRF 180.0
#0
pulse [expr 46.0*$C90/90.0] $CRF 0.0
pulse [expr 96.0*$C90/90.0] $CRF 45.0
pulse [expr 164.0*$C90/90.0] $CRF 67.5
pulse [expr 159.0*$C90/90.0] $CRF 315.0
pulse [expr 130.0*$C90/90.0] $CRF 22.5
pulse [expr 159.0*$C90/90.0] $CRF 315.0
pulse [expr 164.0*$C90/90.0] $CRF 67.5
pulse [expr 96.0*$C90/90.0] $CRF 45.0
pulse [expr 46.0*$C90/90.0] $CRF 0.0
#0
pulse [expr 46.0*$C90/90.0] $CRF 0.0
pulse [expr 96.0*$C90/90.0] $CRF 45.0
pulse [expr 164.0*$C90/90.0] $CRF 67.5
pulse [expr 159.0*$C90/90.0] $CRF 315.0
pulse [expr 130.0*$C90/90.0] $CRF 22.5
pulse [expr 159.0*$C90/90.0] $CRF 315.0
pulse [expr 164.0*$C90/90.0] $CRF 67.5
pulse [expr 96.0*$C90/90.0] $CRF 45.0
pulse [expr 46.0*$C90/90.0] $CRF 0.0
```

```
#0
pulse [expr 46.0*$C90/90.0] $CRF 0.0
pulse [expr 96.0*$C90/90.0] $CRF 45.0
pulse [expr 164.0*$C90/90.0] $CRF 67.5
pulse [expr 159.0*$C90/90.0] $CRF 315.0
pulse [expr 130.0*$C90/90.0] $CRF 22.5
pulse [expr 159.0*$C90/90.0] $CRF 315.0
pulse [expr 164.0*$C90/90.0] $CRF 67.5
pulse [expr 96.0*$C90/90.0] $CRF 45.0
pulse [expr 46.0*$C90/90.0] $CRF 0.0
#180
pulse [expr 46.0*$C90/90.0] $CRF 180.0
pulse [expr 96.0*$C90/90.0] $CRF 225.0
pulse [expr 164.0*$C90/90.0] $CRF 247.5
pulse [expr 159.0*$C90/90.0] $CRF 495.0
pulse [expr 130.0*$C90/90.0] $CRF 202.5
pulse [expr 159.0*$C90/90.0] $CRF 495.0
pulse [expr 164.0*$C90/90.0] $CRF 247.5
pulse [expr 96.0*$C90/90.0] $CRF 225.0
pulse [expr 46.0*$C90/90.0] $CRF 180.0
#180
pulse [expr 46.0*$C90/90.0] $CRF 180.0
pulse [expr 96.0*$C90/90.0] $CRF 225.0
pulse [expr 164.0*$C90/90.0] $CRF 247.5
pulse [expr 159.0*$C90/90.0] $CRF 495.0
pulse [expr 130.0*$C90/90.0] $CRF 202.5
pulse [expr 159.0*$C90/90.0] $CRF 495.0
pulse [expr 164.0*$C90/90.0] $CRF 247.5
pulse [expr 96.0*$C90/90.0] $CRF 225.0
pulse [expr 46.0*$C90/90.0] $CRF 180.0
#180
pulse [expr 46.0*$C90/90.0] $CRF 180.0
pulse [expr 96.0*$C90/90.0] $CRF 225.0
pulse [expr 164.0*$C90/90.0] $CRF 247.5
pulse [expr 159.0*$C90/90.0] $CRF 495.0
pulse [expr 130.0*$C90/90.0] $CRF 202.5
pulse [expr 159.0*$C90/90.0] $CRF 495.0
pulse [expr 164.0*$C90/90.0] $CRF 247.5
```

```
pulse [expr 96.0*$C90/90.0] $CRF 225.0
pulse [expr 46.0*$C90/90.0] $CRF 180.0
#0
pulse [expr 46.0*$C90/90.0] $CRF 0.0
pulse [expr 96.0*$C90/90.0] $CRF 45.0
pulse [expr 164.0*$C90/90.0] $CRF 67.5
pulse [expr 159.0*$C90/90.0] $CRF 315.0
pulse [expr 130.0*$C90/90.0] $CRF 22.5
pulse [expr 159.0*$C90/90.0] $CRF 315.0
pulse [expr 164.0*$C90/90.0] $CRF 67.5
pulse [expr 96.0*$C90/90.0] $CRF 45.0
pulse [expr 46.0*$C90/90.0] $CRF 0.0
#0
pulse [expr 46.0*$C90/90.0] $CRF 0.0
pulse [expr 96.0*$C90/90.0] $CRF 45.0
pulse [expr 164.0*$C90/90.0] $CRF 67.5
pulse [expr 159.0*$C90/90.0] $CRF 315.0
pulse [expr 130.0*$C90/90.0] $CRF 22.5
pulse [expr 159.0*$C90/90.0] $CRF 315.0
pulse [expr 164.0*$C90/90.0] $CRF 67.5
pulse [expr 96.0*$C90/90.0] $CRF 45.0
pulse [expr 46.0*$C90/90.0] $CRF 0.0
#180
pulse [expr 46.0*$C90/90.0] $CRF 180.0
pulse [expr 96.0*$C90/90.0] $CRF 225.0
pulse [expr 164.0*$C90/90.0] $CRF 247.5
pulse [expr 159.0*$C90/90.0] $CRF 495.0
pulse [expr 130.0*$C90/90.0] $CRF 202.5
pulse [expr 159.0*$C90/90.0] $CRF 495.0
pulse [expr 164.0*$C90/90.0] $CRF 247.5
pulse [expr 96.0*$C90/90.0] $CRF 225.0
pulse [expr 46.0*$C90/90.0] $CRF 180.0
#180
pulse [expr 46.0*$C90/90.0] $CRF 180.0
pulse [expr 96.0*$C90/90.0] $CRF 225.0
pulse [expr 164.0*$C90/90.0] $CRF 247.5
pulse [expr 159.0*$C90/90.0] $CRF 495.0
pulse [expr 130.0*$C90/90.0] $CRF 202.5
```

```
pulse [expr 159.0*$C90/90.0] $CRF 495.0
pulse [expr 164.0*$C90/90.0] $CRF 247.5
pulse [expr 96.0*$C90/90.0] $CRF 225.0
pulse [expr 46.0*$C90/90.0] $CRF 180.0
#180
pulse [expr 46.0*$C90/90.0] $CRF 180.0
pulse [expr 96.0*$C90/90.0] $CRF 225.0
pulse [expr 164.0*$C90/90.0] $CRF 247.5
pulse [expr 159.0*$C90/90.0] $CRF 495.0
pulse [expr 130.0*$C90/90.0] $CRF 202.5
pulse [expr 159.0*$C90/90.0] $CRF 495.0
pulse [expr 164.0*$C90/90.0] $CRF 247.5
pulse [expr 96.0*$C90/90.0] $CRF 225.0
pulse [expr 46.0*$C90/90.0] $CRF 180.0
#0
pulse [expr 46.0*$C90/90.0] $CRF 0.0
pulse [expr 96.0*$C90/90.0] $CRF 45.0
pulse [expr 164.0*$C90/90.0] $CRF 67.5
pulse [expr 159.0*$C90/90.0] $CRF 315.0
pulse [expr 130.0*$C90/90.0] $CRF 22.5
pulse [expr 159.0*$C90/90.0] $CRF 315.0
pulse [expr 164.0*$C90/90.0] $CRF 67.5
pulse [expr 96.0*$C90/90.0] $CRF 45.0
pulse [expr 46.0*$C90/90.0] $CRF 0.0
#0
pulse [expr 46.0*$C90/90.0] $CRF 0.0
pulse [expr 96.0*$C90/90.0] $CRF 45.0
pulse [expr 164.0*$C90/90.0] $CRF 67.5
pulse [expr 159.0*$C90/90.0] $CRF 315.0
pulse [expr 130.0*$C90/90.0] $CRF 22.5
pulse [expr 159.0*$C90/90.0] $CRF 315.0
pulse [expr 164.0*$C90/90.0] $CRF 67.5
pulse [expr 96.0*$C90/90.0] $CRF 45.0
pulse [expr 46.0*$C90/90.0] $CRF 0.0

store 2
  for {set i 1} {$i<=$par(number_of_spin)} {incr i} {
      matrix set start operator I${i}z
```

```
        reset
        prop 2
#ACQUISITIONn
        for {set j 1} {$j<=$par(number_of_spin)} {incr j} {
           matrix set detect operator I${j}z
           acq
           #puts "j=$j i=$i k=$m"
           }
        }
}
}

proc main {} {
global par
foreach par(flag_no_HC_D) {0 1} {
foreach par(flag_no_HC_J) {0} {
foreach par(flag_no_CC_D) {0 1} {
foreach par(flag_no_CC_J) {0 1} {
foreach par(flag_no_HH_D) {0} {
foreach CSAp {0 30 60} {
foreach C_offset_p {40.7} {
set par(C_offset) [expr $C_offset_p*[resfreq 13C $par(proton_frequency)]/1.0e6]
set CSA [expr $CSAp*[resfreq 13C $par(proton_frequency)]/1.0e6]
set par(out_factor) [expr $par(number_of_spin)**2]
set par(out_factor) [expr $par(number_of_spin)**2]
set nnp 256
set par(np) [expr $par(out_factor)*$nnp]
foreach par(RF_frac_RO) {0.25} {
set scale [expr 16.0*1060.0/90.0]
set File_name {}
for {set i 0} {$i<$par(number_of_spin)} {incr i} {
for {set j 0} {$j<$par(number_of_spin)} {incr j} {
   set operator_name "$par(name)-buildup_RFratio-[expr $par(RF_frac_RO)]-Sta-I[expr
$i+1]z_Det-I[expr $j+1]-CSA_C-[expr $CSAp]-INTERACTION-noHC_D-[expr
$par(flag_no_HC_D)]-noHC_J-[expr $par(flag_no_HC_J)]-noCC_D-[expr
$par(flag_no_CC_D)]-noCC_J-[expr $par(flag_no_CC_J)]-noHH_D-[expr
$par(flag_no_HH_D)].res"
   set current_operator [open "$operator_name" w]
```

```
   lappend File_name $current_operator
}
}
#set f [fsimpson]
set f [fsimpson [list [list shift_1_aniso [expr $CSA]] [list shift_2_aniso [expr $CSA/3.0]]]]
#fsave $f  "$par(name)-buildup_N-$par(N)-n-$par(n)-v-$par(v).fid"
set c 0
#Build Up curve increments
        for {set l 1} {$l <= $nnp} {incr l} {
        set a 0
           for {set i 1} {$i<=$par(number_of_spin)} {incr i} {
                for {set j 1} {$j<=$par(number_of_spin)} {incr j} {
                incr c
                set Sr [findex $f $c -re]
                set Si [findex $f $c -im]
                ##data organized as:
                #col 1: number of full FLOPSY cycles per invividual rec.block
                #col 2: not important
                #col 3: length of recoupling [ms]
                #col 4: Re int
                #col 5: Im int.
                #col 6: Abs int.
                puts [lindex $File_name $a] "$l [expr $l*$scale] [expr
$scale*1.0e3*$l/$par(spin_rate)] [expr $Sr] [expr $Si] [expr sqrt($Sr**2+$Si**2)]"
                incr a
        }
        }
        }
        funload $f
        #close $FileRe_Im_Abs
        for {set p 0} {$p<$par(out_factor)} {incr p} {
        close [lindex $File_name $p]
        }
}
}
}
}}}}}
}
```

1.2.2 $^{13}C_6$ spin system

```
#SIMPSON script for CN^v_n 13C-13C recoupling simulation in a specific magnetic field
#Copyright Piotr Paluch CNBCh University of Warsaw 2020
#CN^v_n buildup
#For 6 spin system containing only 13C.
#Coordinates taken from PDB SH3.
#Dipol-dipol interaction strengths calculated in simmol.
#Chemical shift parameters taken from microcrystalline fMLF.
#CSA parameters estimated using Gaussian.
#crystal file rep168, gamma angle 11 (checked for convergence).
#For every N, n, v, the script generates K^2 files where K is the spin system size.
#1H basis frequency (possible multiple in the foreach loop), N, n, v and carrier frequency
# are set in main section.
#RF field during recoupling is set automatically based on spinning speed.

spinsys {
#     1     2     3     4     5     6
#   20CA   21C   23CB   24CG   25CD1   26CD2
#
channels 13C
nuclei   13C 13C 13C 13C 13C 13C

shift 1 56.8p -30.0p 0.89 0 0 0
#CO
shift 2 175.0p 124.3p 0.99 0 0 0
#CB
shift 3 40.6p 25.9p 0.98 0 0 0
#CC
shift 4 25.0p 9.6p 0.70 0 0 0
#CD
shift 5 24.6p -13.3p 0.29 0 0 0
#CD2
shift 6 19.6p -10.3p 0.20 0 0 0

dipole 1 2 -2180.16 0 131.27 149.11
dipole 1 3 -2084.9 0 92.756 -93.655
dipole 1 4 -472.29 0 66.144 -71.62
```

```
dipole 1 5 -131.28 0 76.089 -81.145
dipole 1 6 -302.44 0 74.026 -39.394

dipole 2 3 -501.62 0 68.022 -67.447
dipole 2 4 -134.8 0 58.19 -58.427
dipole 2 5 -62.301 0 67.096 -70.085
dipole 2 6 -92.948 0 65.41 -36.945

dipole 3 4 -2160.1 0 43.946 -38.499
dipole 3 5 -492.19 0 66.221 -72.742
dipole 3 6 -515.77 0 68.963 -6.3485

dipole 4 5 -2125.2 0 93.412 -95.64
dipole 4 6 -2169.6 0 98.139 15.589

dipole 5 6 -484.32 0 92.839 50.274

jcoupling 1 2 50.0 0 0 0 0 0
jcoupling 1 3 33.0 0 0 0 0 0
jcoupling 1 4 3.0 0 0 0 0 0

jcoupling 2 3 3.0 0 0 0 0 0

jcoupling 3 4 33.0 0 0 0 0 0
jcoupling 3 5 3.0 0 0 0 0 0
jcoupling 3 6 3.0 0 0 0 0 0

jcoupling 4 5 33.0 0 0 0 0 0
jcoupling 4 6 33.0 0 0 0 0 0

jcoupling 5 6 3.0 0 0 0 0 0
}

par {
method dsyev
conjugate_fid false
   proton_frequency 600e6
   spin_rate        100000.0
```

```
    np                              1
    crystal_file           rep168
    gamma_angles                 11
    start_operator               I1z
    detect_operator              I2z
    verbose                         1101
    # Spin system size
    variable number_of_spin 6
    #C sequence param
    # they will be changed according to NN, nn, vv values in the main section
    #n: number of rotor steps for a full cycle
    #N: number of cycles for n rotor periods
    #v: number of full phase evolutions for n rotor periods
    variable N 0.0
    variable n 0.0
    variable v 0.0
    #####
    variable out_factor 1
    variable C_offset 0.0
    ######
}

proc pulseq {} {
global par
maxdt 0.1
set Taur        [expr 1.0e6/$par(spin_rate)]
set C90_CN  [expr $Taur*$par(n)/(8.0*$par(N))]
set Phase_increment [expr 360.0*$par(v)/(1.0*$par(N))]
set CN_RF [expr 0.25e6/$C90_CN]

    #Excitation buildup complete cycle
reset
    for {set k 0} {$k<$par(N)} {incr k} {
    offset $par(C_offset)
    pulse $C90_CN $CN_RF [expr $k*$Phase_increment]
    pulse [expr 4.0*$C90_CN]  $CN_RF [expr 180.0+$k*$Phase_increment]
    pulse [expr 3.0*$C90_CN]  $CN_RF [expr $k*$Phase_increment]
    }
```

```
store 1

reset
store 2

for {set m 1} {$m<=[expr $par(np)/$par(out_factor)]} {incr m} {
    reset
    prop 2
    prop 1
    store 2
    for {set i 1} {$i<=$par(number_of_spin)} {incr i} {
          matrix set start operator I${i}z
          reset
          prop 2
#ACQUISITIONn
          for {set j 1} {$j<=$par(number_of_spin)} {incr j} {
              matrix set detect operator I${j}z
              acq
              #puts "j=$j i=$i k=$m"
              }
          }
}
}

proc main {} {
global par
foreach 1H_freq_MHz {800.0} {
set par(proton_frequency) [expr 1.0e6*$1H_freq_MHz]
foreach C_offset_p {36.0} {
#set par(C_offset) [expr (62.50-37.73)*[resfreq 13C $par(proton_frequency)]/1.0e6]
set par(C_offset) [expr $C_offset_p*[resfreq 13C $par(proton_frequency)]/1.0e6]
## Here N,n,v value are given as NN,nn,vv
foreach NN {9 9 9 9 9 9} nn {24 30 33 39 42 48} vv {1 1 1 1 1 1 1 1 1 1 1} nnp {300 240 220
184 180 160} {
set par(N) $NN
set par(n) $nn
set par(v) $vv
set par(out_factor) [expr $par(number_of_spin)**2]
```

```
set par(np) [expr $par(out_factor)*$nnp]

set File_name {}
for {set i 0} {$i<$par(number_of_spin)} {incr i} {
for {set j 0} {$j<$par(number_of_spin)} {incr j} {
   set operator_name "$par(name)-buildup_N-$par(N)-n-$par(n)-v-$par(v)_Start-I[expr
$i+1]z_Detect-I[expr $j+1]z_C_offset-[expr $C_offset_p]ppm_1H_Freq-[expr
$1H_freq_MHz]MHz.res"
   set current_operator [open "$operator_name" w]
   lappend File_name $current_operator
}
}

set f [fsimpson]
set c 0
#Build Up curve increments
        for {set l 1} {$l <= $nnp} {incr l} {
        set a 0
           for {set i 1} {$i<=$par(number_of_spin)} {incr i} {
                for {set j 1} {$j<=$par(number_of_spin)} {incr j} {
                incr c
                set Sr [findex $f $c -re]
                set Si [findex $f $c -im]
                ##data organized as:
                #col 1: number of full CN_n^v cycles per invividual rec.block
                #col 2: length of recoupling [ms]
                #col 3: Re int
                #col 4: Im int.
                #col 5: Abs int.
                puts [lindex $File_name $a] "$l [expr $nn*1.0e3*$l/$par(spin_rate)] [expr $Sr]
[expr $Si] [expr sqrt($Sr**2+$Si**2)]"
                incr a
        }
        }
        }
        funload $f
        #close $FileRe_Im_Abs
        for {set p 0} {$p<$par(out_factor)} {incr p} {
```

```
        close [lindex $File_name $p]
        }
}
}
}
}
```

1.3 Calculation of transfer efficiency in fMLF spectra

Absolute $C\delta_1 \rightarrow C\alpha$ transfer efficiencies in spectra of fMLF were obtained as follows. We first express the intensities of a diagonal (δ_1, δ_1) peak at beginning of the series ($\tau_{MIX} = 0$), $S_0^{2D}(\delta, \delta)$, and the intensity of an α-detected cross-peak (δ_1, α) at nonzero mixing, $S_\tau^{2D}(\delta, \alpha)$:

$$S_0^{2D}(\delta, \delta) = I_\delta^0 \kappa_1(\delta) \kappa_2(\delta) A$$

$$S_\tau^{2D}(\delta, \alpha) = \varepsilon\, I_\delta^0 \kappa_1(\delta) \kappa_2(\alpha) A,$$

where I_δ^0 stands for initial polarization of $^{13}C\delta_1$, ε is the mixing efficiency for given τ_{MIX}, A is a constant related to 2D acquisition, and $\kappa_1(\delta)$ and $\kappa_2(\delta)$ are integral factors originating from chemical shift evolution of $^{13}C\delta_1$ chemical shift in the indirect and direct dimensions, respectively (and similarly for $^{13}C\alpha$ chemical shift evolution). Elimination of the $\kappa_2(\delta)/\kappa_2(\alpha)$ factor is required to determine transfer efficiency ε.

To this end, 1D CP ^{13}C detected experiments are recorded with the exactly same parameters as the 2D mixing series (recycling delay, CP powers, ^{13}C directly detected FID sampling and processing). Peak intensities are given by:

$S^{1D}(\delta) = I_\delta^0 \kappa_2(\delta)$, and similarly,

$$S^{1D}(\alpha) = I_\alpha^0 \kappa_2(\alpha)$$

In contrast to peak intensity, its integral (I_δ^{1D} or I_α^{1D}) is independent of the line-width (or shape), and proportional to initial polarization I_δ^0 (or I_α^0). We thus establish that

$$\frac{I_\alpha^0}{I_\delta^0} = \frac{I_\alpha^{1D}}{I_\delta^{1D}}$$

and find that:

$$\varepsilon = \frac{S_\tau^{2D}(\delta, \alpha)}{S_0^{2D}(\delta, \delta)} \frac{\kappa_2(\delta)}{\kappa_2(\alpha)} = \frac{S_\tau^{2D}(\delta, \alpha)}{S_0^{2D}(\delta, \delta)} \frac{S^{1D}(\delta)}{S^{1D}(\alpha)} \frac{I_\alpha^0}{I_\delta^0} = \frac{S_\tau^{2D}(\delta, \alpha)}{S_0^{2D}(\delta, \delta)} \frac{S^{1D}(\delta)}{S^{1D}(\alpha)} \frac{I_\alpha^{1D}}{I_\delta^{1D}}$$

It is thus sufficient to evaluate relative intensities and integrals of $^{13}C\delta_1$ and $^{13}C\alpha$ peaks in the 1D spectrum, and relate the cross-peak intensity (at τ_{MIX}) to the diagonal one (at $\tau_{MIX} = 0$).

2 Supplementary Figures

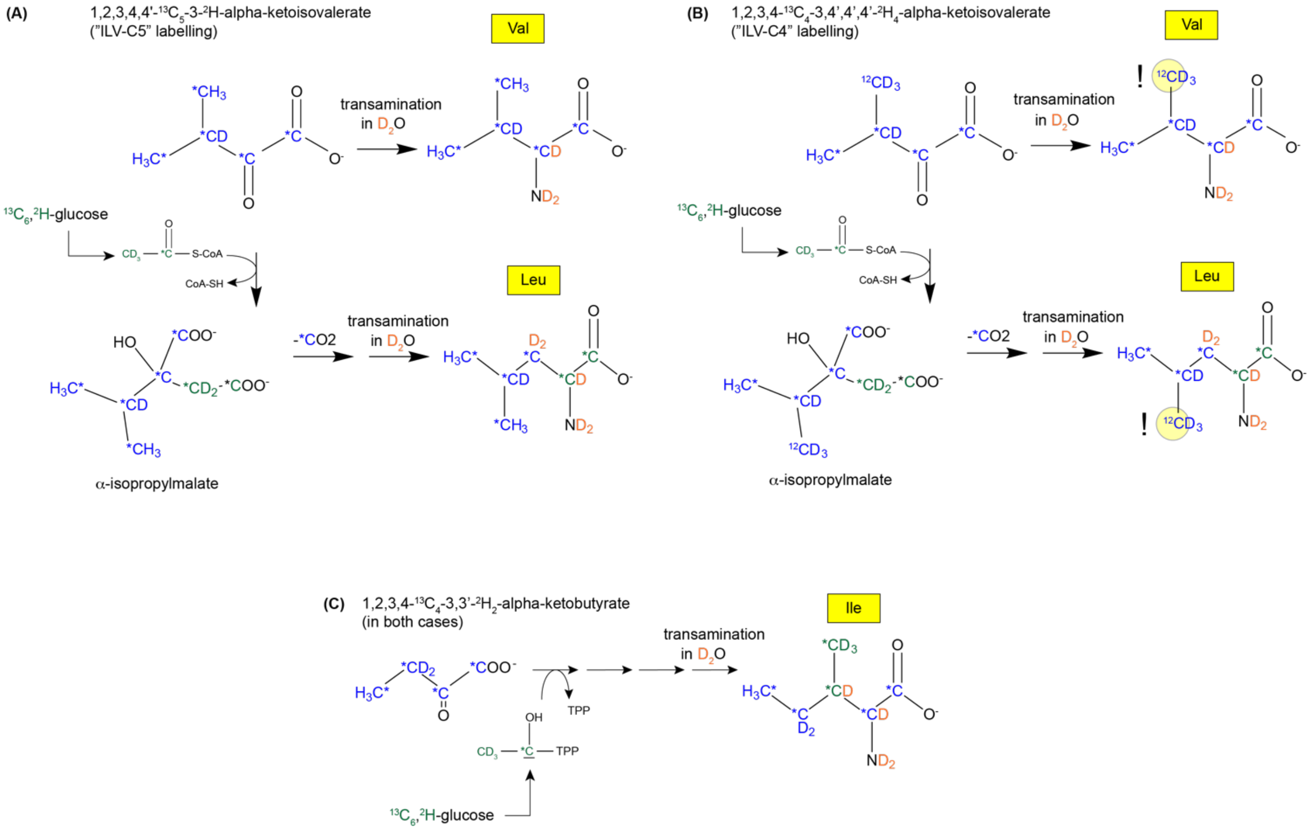

Supplementary Figure S1. ^{13}C and ^{2}H isotopic enrichment patterns of Valine and Leucine residues obtained using (A) uniformly ^{13}C-labelled, and (B) non-stereospecifically $^{12}CD_3/^{13}CH_3$-labelled (otherwise ^{13}C-labelled) alpha-ketoisovalerate, and ^{13}C, ^{2}H-enriched glucose. (C) Isotropic enrichment pattern for Isoleucine residues obtained from uniformly ^{13}C, ^{2}H-enriched alpha-ketobutyrate, and ^{13}C, ^{2}H-enriched glucose. Patterns predicted on the basis of biosynthetic pathways presented by Lundström et al. (Journal of Biomolecular NMR, 38, 199-212 (2007)).

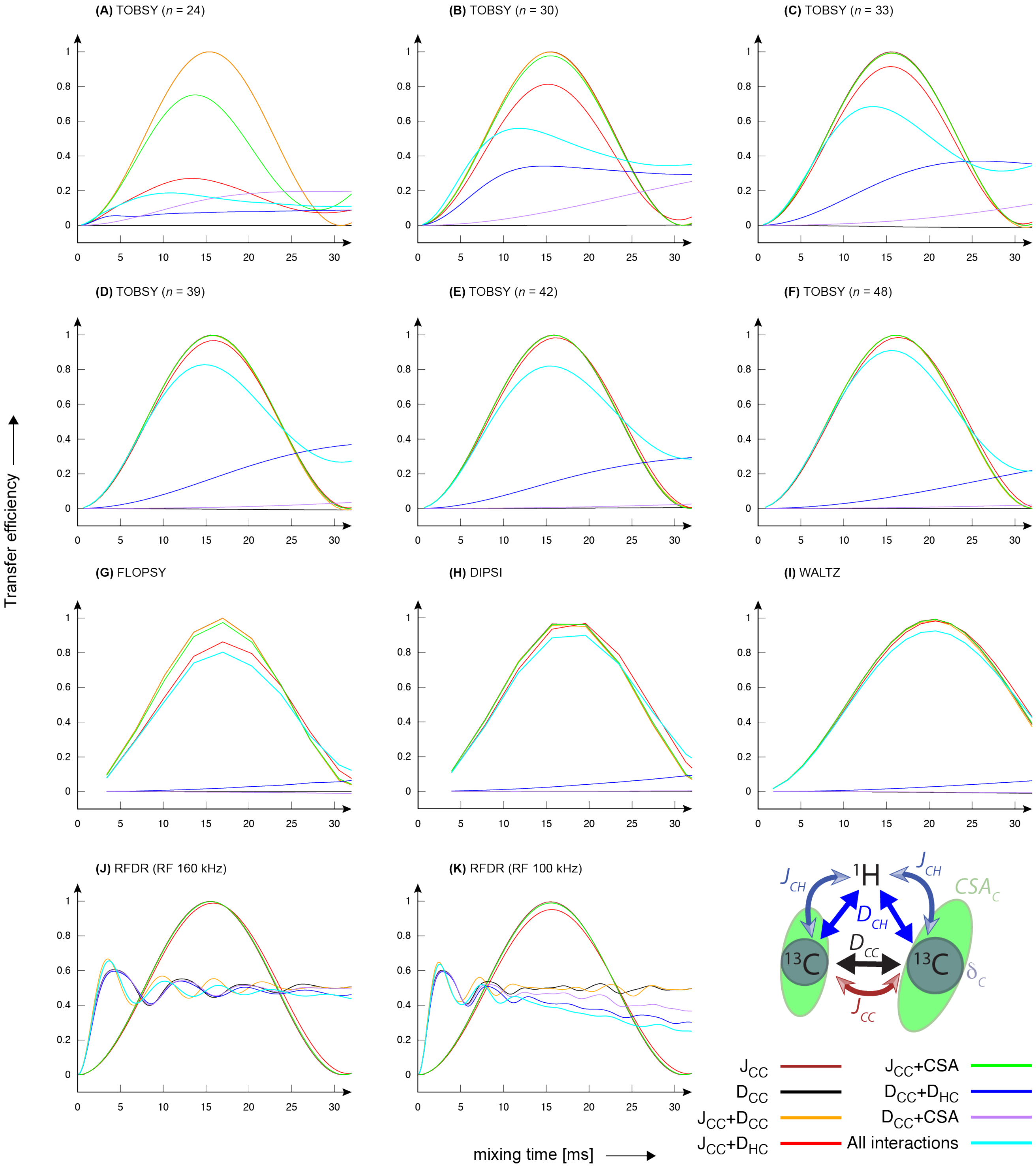

Supplementary Figure S2. $^{13}C\beta \rightarrow {}^{13}C\alpha$ coherence transfer efficiency simulated in SIMPSON for a C_2H_2 spin system mimicking an alanine residue. For a specific ^{13}C mixing, individual isotropic (J_{CC}, CS) and anisotropic spin interactions (D_{CC}, D_{HC}, ^{13}C CSA) were enabled and disabled, and relevant pairs of interactions selected (color-coded curves). A total effect of mixing in presence of all interactions is indicated by cyan curves. (A-F) show TOBSY $C9^1_n$ with $n = 24$, 30, 33, 39, 42 and 48, (H-J) show TOCSY mixing (FLOPSY-16, DIPSI-3 and WALTZ, respectively), and (K, L) show

RFDR at $\nu_{1,C}$ = 160 kHz and 100 kHz, respectively. MAS frequency of 55.5 kHz, and B_0 field strength of 18.8 T were assumed.

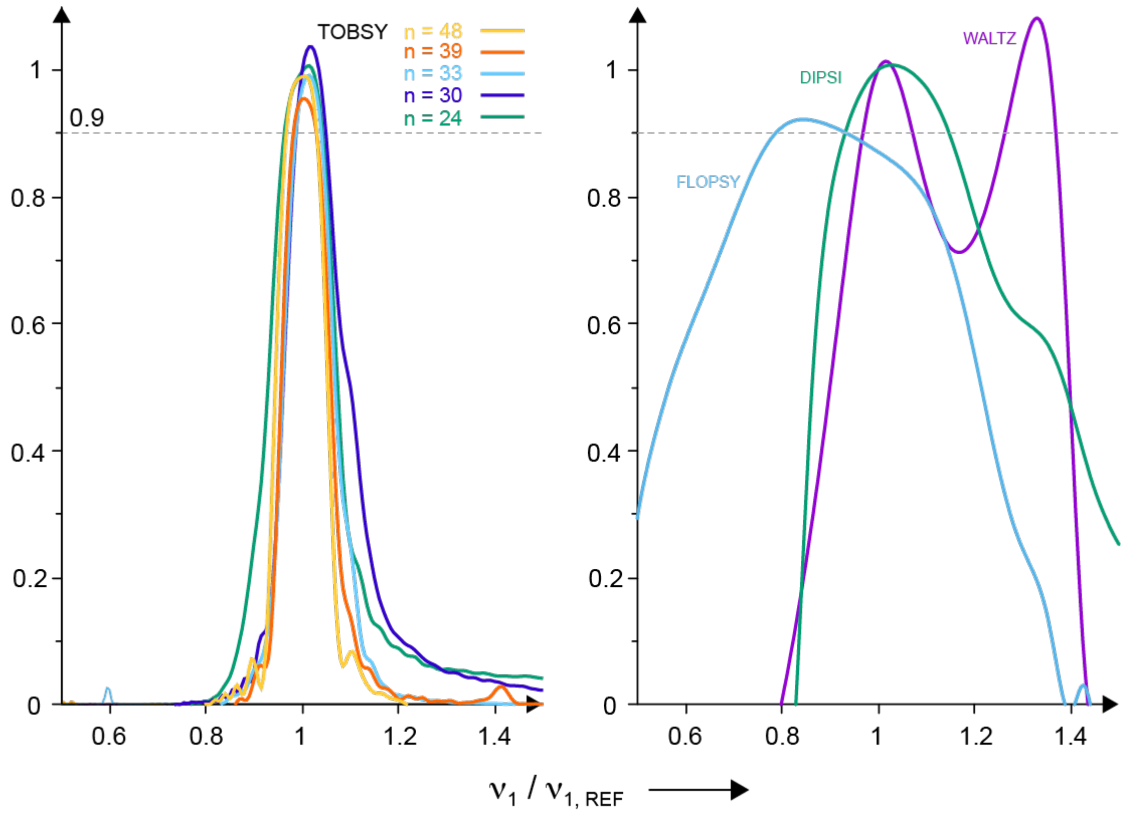

Supplementary Figure S3. Effect of RF miscalibration on the efficiency of TOBSY (left panel) and TOCSY (right panel) ^{13}C mixing schemes, simulated for C_2H_2 spin system as described in caption to Supplementary Figure S2, at the same ν_R and B_0 field strength conditions. Quality threshold of 0.9 is marked with a dashed line.

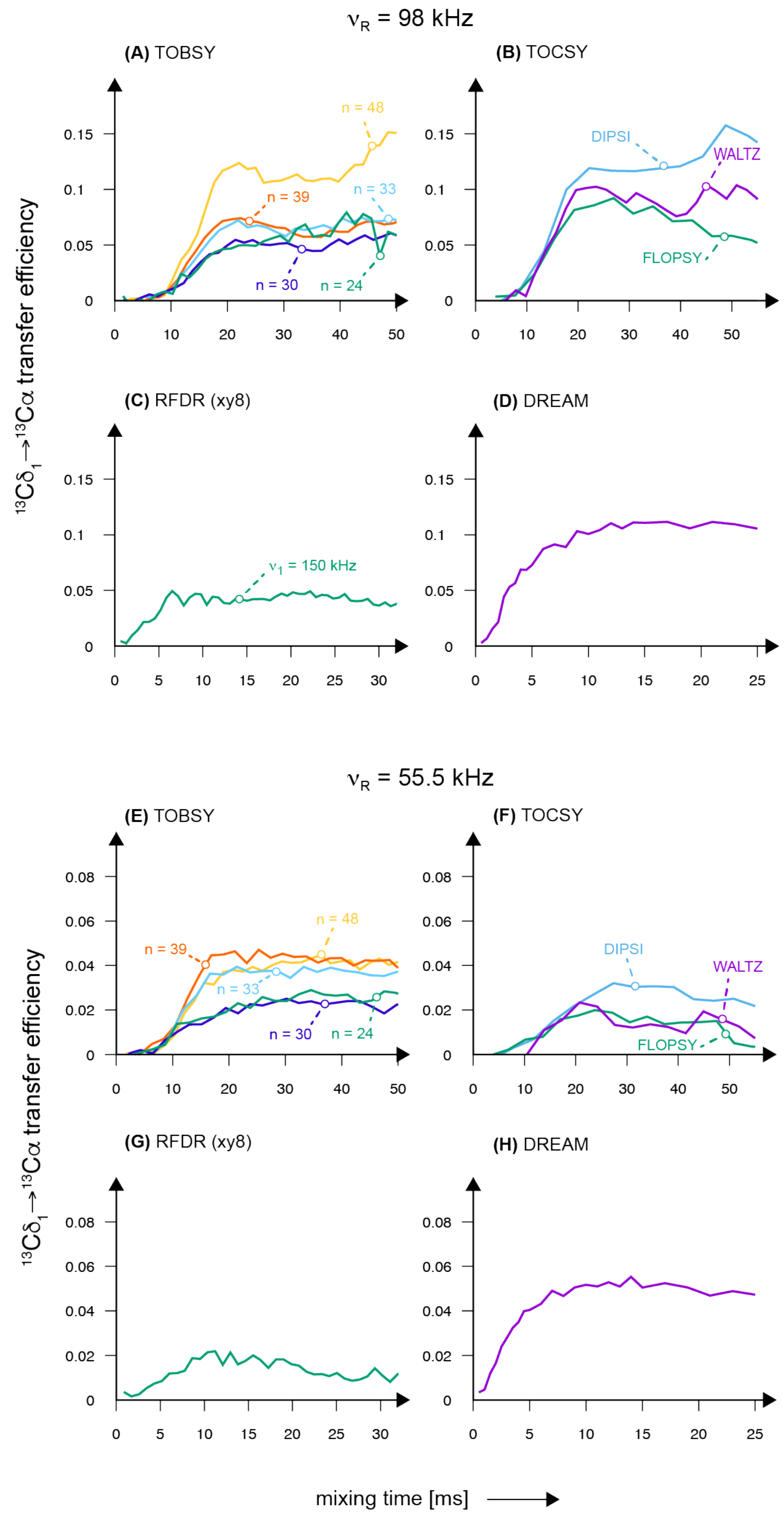

Supplementary Figure S4. Experimentally determined efficiency of $C\delta_1 \rightarrow C\alpha$ transfer in leucine residue of fMLF in function of mixing time (build-up) at $B_0 = 18.8$ T field strength and at $\nu_R = 98$ kHz (*top* panels A-D) and 55.5 kHz (*bottom* panels E-F). Note the efficiency scale difference between $\nu_R = 55.5$ and 98 kHz cases. Actual S/N (normalized to 24 h of acquisition) can be obtained by multiplication of data by 6646 and 4886 for data recorded at $\nu_R = 55.5$ and 98 kHz, respectively. CP efficiency was lower by a factor of 1.35 at $\nu_R = 98$ kHz.

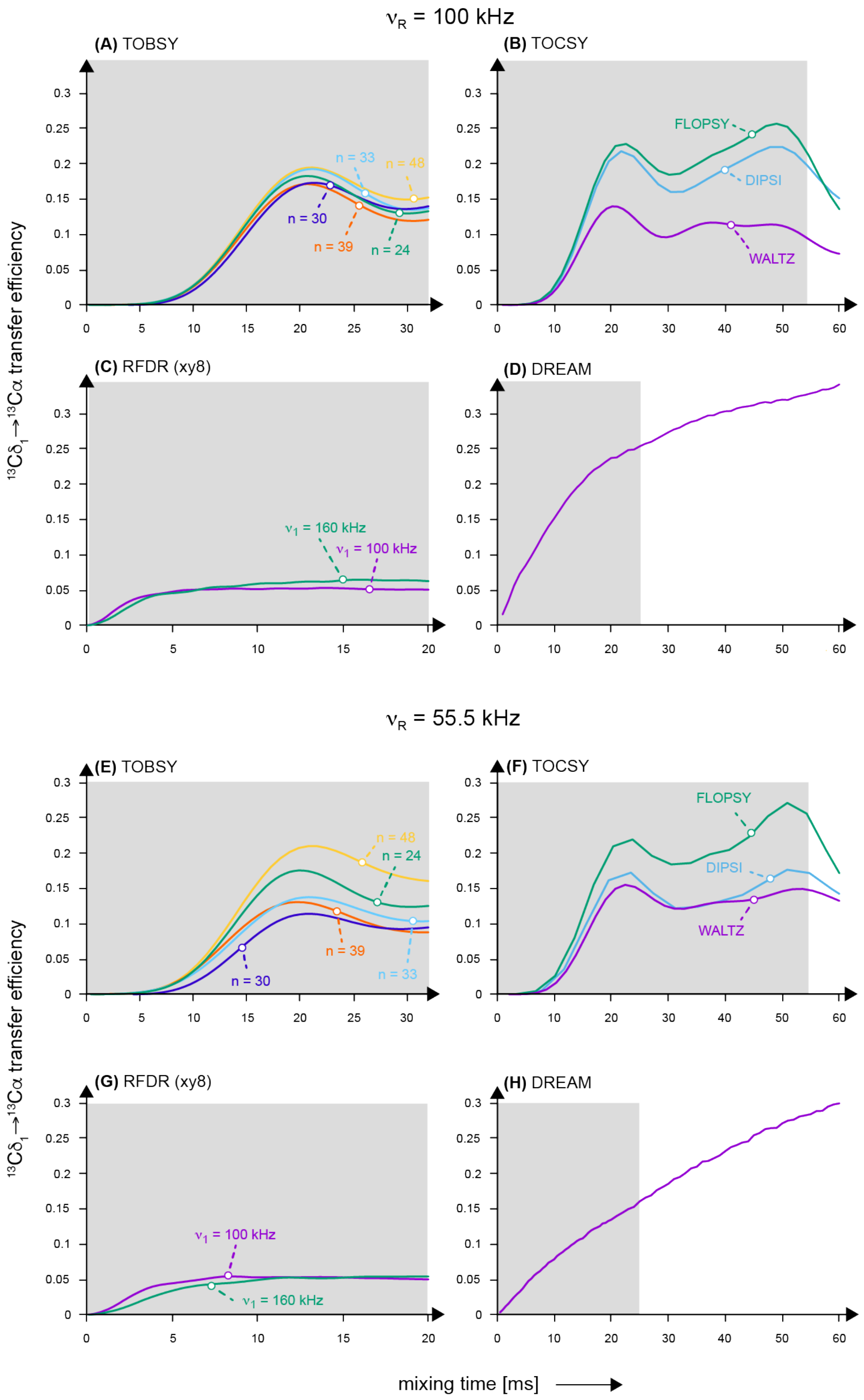

Supplementary Figure S5. $C\delta_1 \rightarrow C\alpha$ transfer efficiency in function of mixing time, simulated in SIMPSON for a Leucine-like $^{13}C_6$-spin system for $B_0 = 18.8$ T field strength and at $\nu_R = 100$ kHz (*top* panels A-D) and 55.5 kHz (*bottom* panels E-F). Grey boxes indicate the mixing time ranges sampled experimentally for fMLF sample.

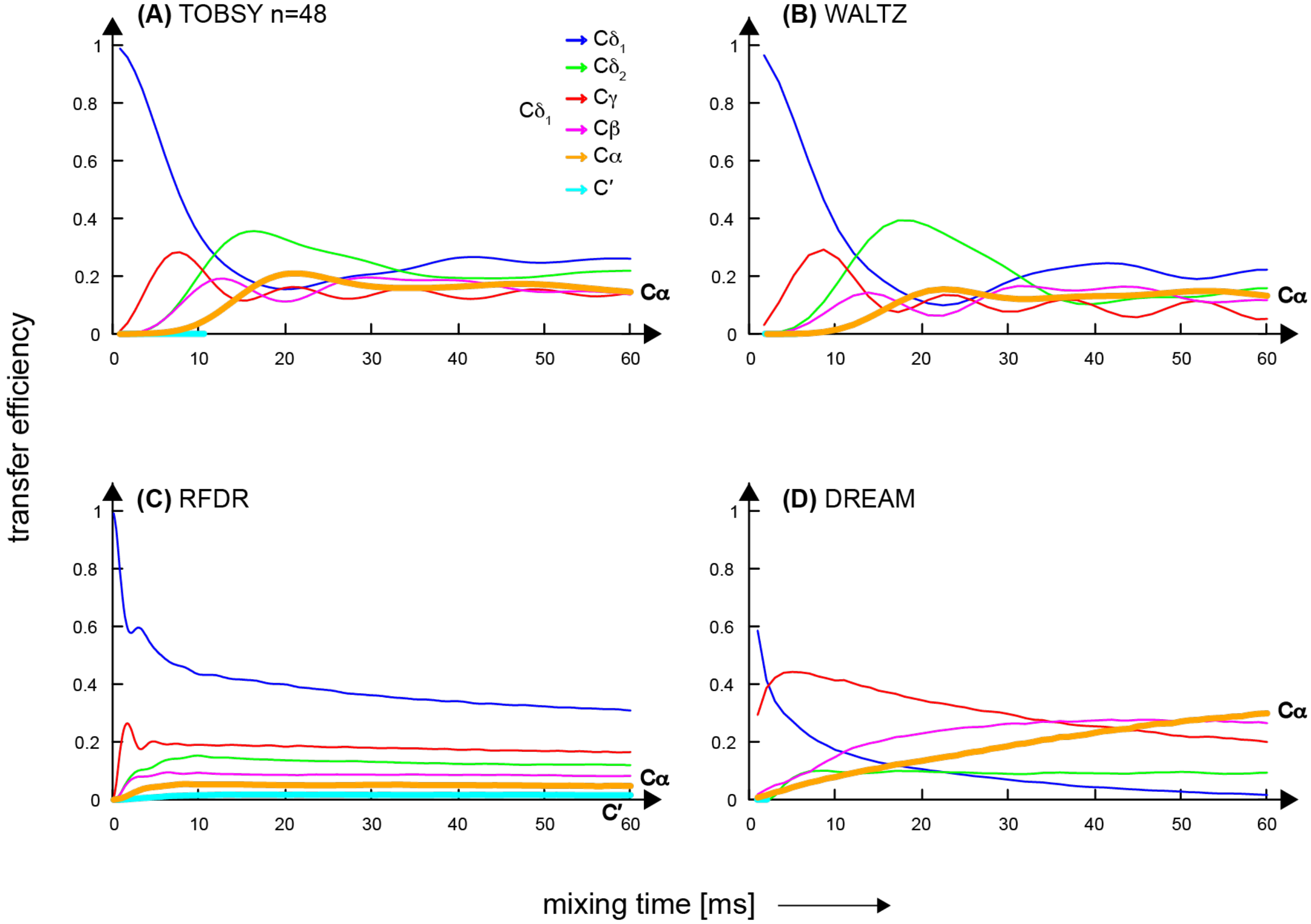

Supplementary Figure S6. Efficiency of coherence transfer from Cδ$_1$ to all other carbon spins in a Leucine-like $^{13}C_6$ spin system in function of mixing time, simulated in SIMPSON for B_0 = 18.8 T field strength and at ν_R = 55.5 kHz. RF schemes based on recoupling of isotropic J_{CC} interaction show oscillatory behavior (A, B), while sequences intended to recouple anisotropic D_{CC} show smooth dependencies.

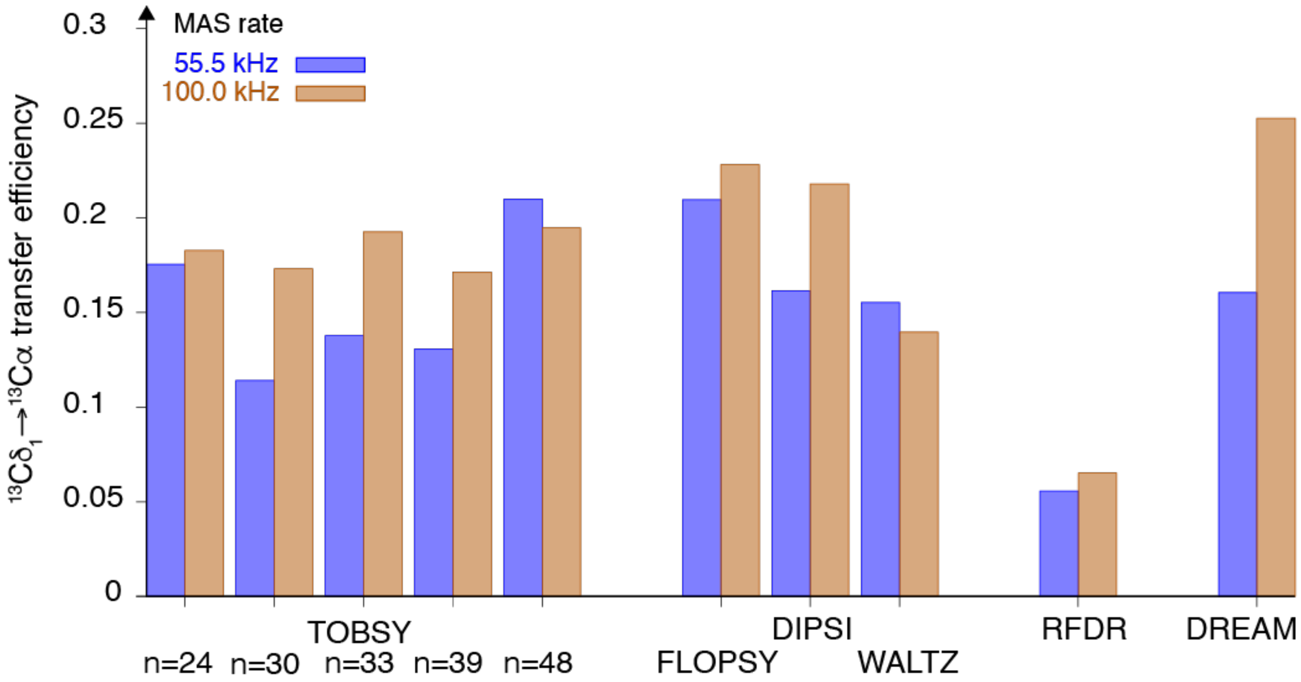

Supplementary Figure S7. Maximum $C\delta_1 \rightarrow C\alpha$ transfer efficiency (at optimal mixing times for each case) in $^{13}C_6$ spin system simulated in SIMPSON for $B_0 = 18.8$ T field and at $\nu_R = 55.5$ (*blue* bars, left in each pair) and 100 kHz (*tan* bars, right in each pair). For DREAM, efficiencies at $\tau_{MIX} = 25$ ms (the largest mixing time sampled experimentally on fMLF) were included for this comparison due to absence of an optimum for this mixing.

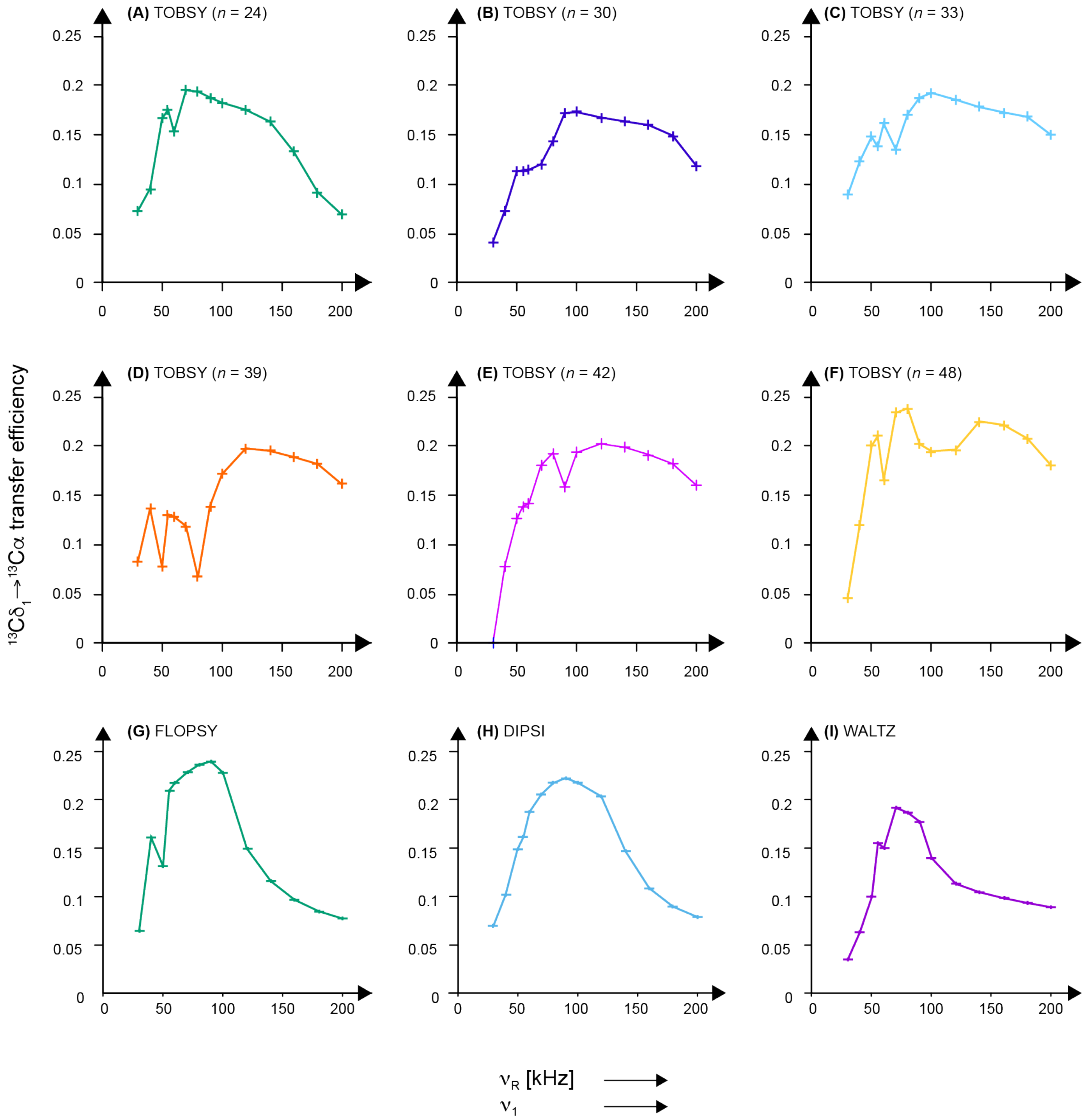

Supplementary Figure S8. Maximum attainable $C\delta_1 \rightarrow C\alpha$ transfer efficiency in a Leucine-like $^{13}C_6$ spin system in function of MAS frequency ν_R, simulated in SIMPSON for $B_0 = 18.8$ T field strength. For the mixing schemes shown, RF strength ν_1 remained in a constant proportion (1:4) to ν_R. For each rotation condition, a full buildup was simulated, and the optimal mixing time selected. RFDR is omitted in this comparison since variable rotation frequency would entail an unintended modulation of rotor filling factor by π pulses (for a constant RF strength ν_1).

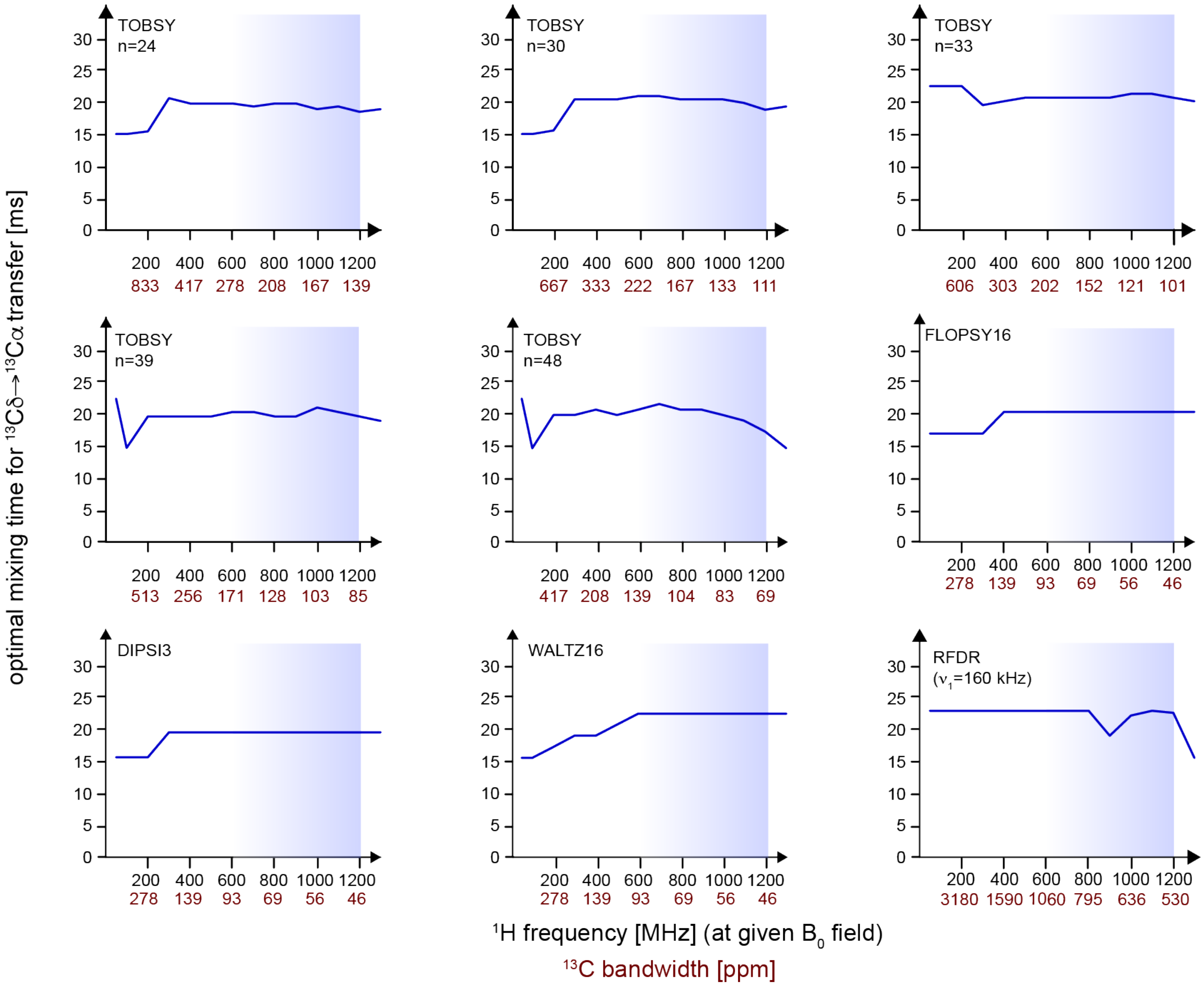

Supplementary Figure S9. Optimal mixing time for $C\delta_1 \rightarrow C\alpha$ transfer in a Leucine-like $^{13}C_6$ spin system simulated in SIMPSON for a range of magnetic fields B_0 (corresponding to ^1H frequency between 50 and 1300 MHz), MAS frequency of 55.5 kHz and a constant RF strength of $\nu_1 = \frac{1}{4}\,\nu_R \approx$ 13.9 kHz. A minimum step for mixing time (discretization) is due to the length of a primitive phase-cycled block of a specific RF scheme.

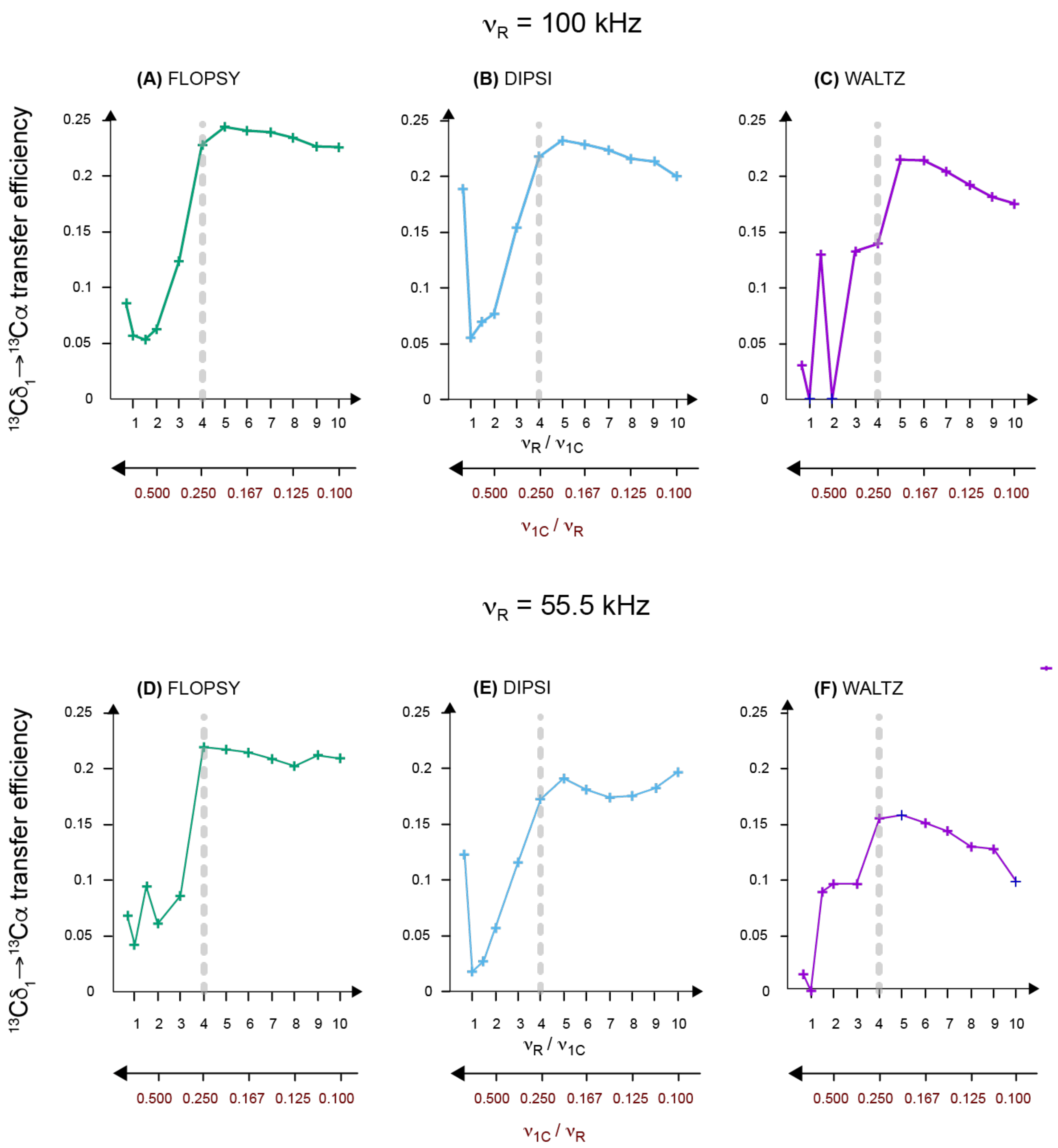

Supplementary Figure S10. $C\delta_1 \rightarrow C\alpha$ transfer efficiency in a Leucine-like $^{13}C_6$ spin system in function of RF strength ν_1, at constant MAS frequency of (A-C) $\nu_R = 100$ kHz and (D-F) 55.5 kHz simulated in SIMPSON for $B_0 = 18.8$ T field. For each RF strength, the value at the optimal mixing time in an individual buildup curve is presented. For all presented TOCSY sequences, destructive recoupling conditions are mostly avoided below $\nu_1 < \frac{1}{4}\,\nu_R$.

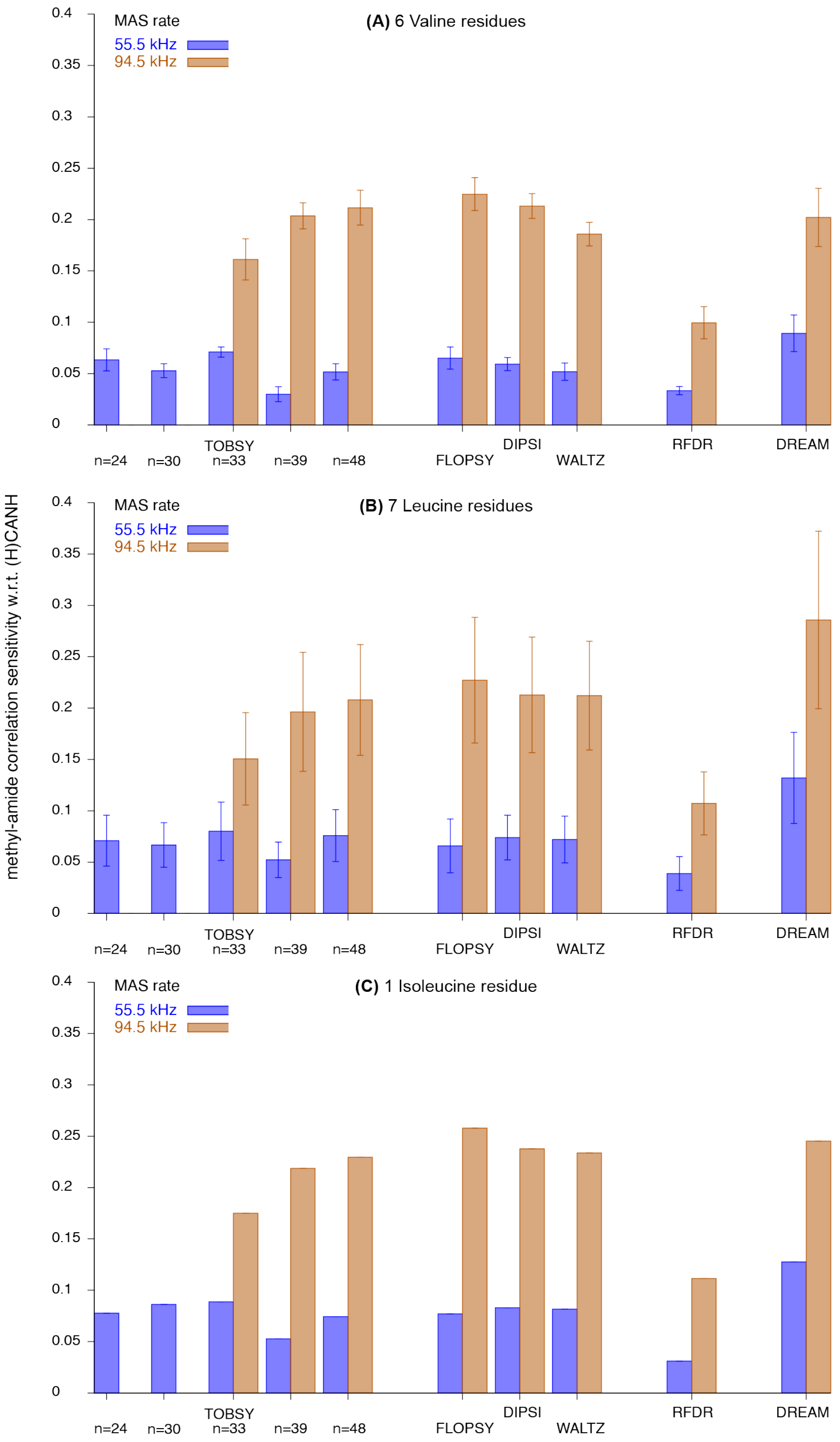

Supplementary Figure S11. Sensitivity of methyl-to-amide correlations in 3D (H)C(CC)(CA)NH spectra normalized to sensitivity of respective intraresidue correlations in 3D (H)CANH spectra, recorded for SH3 protein at MAS frequency of 55.5 (*blue* bars, left in each pair) and 94.5 kHz (*tan* bars, right in each pair) on an 18.8 T spectrometer using various ^{13}C mixing schemes. Relative intensity was averaged for (A) 6 valine (all except Val-46), and (B) 6 leucine residues. (C) presents the comparison for isoleucine-30 residue. In (A, B) the error bars reflect the standard deviation (scatter) of values observed for a set of residues, not the experimental error of average sensitivity.

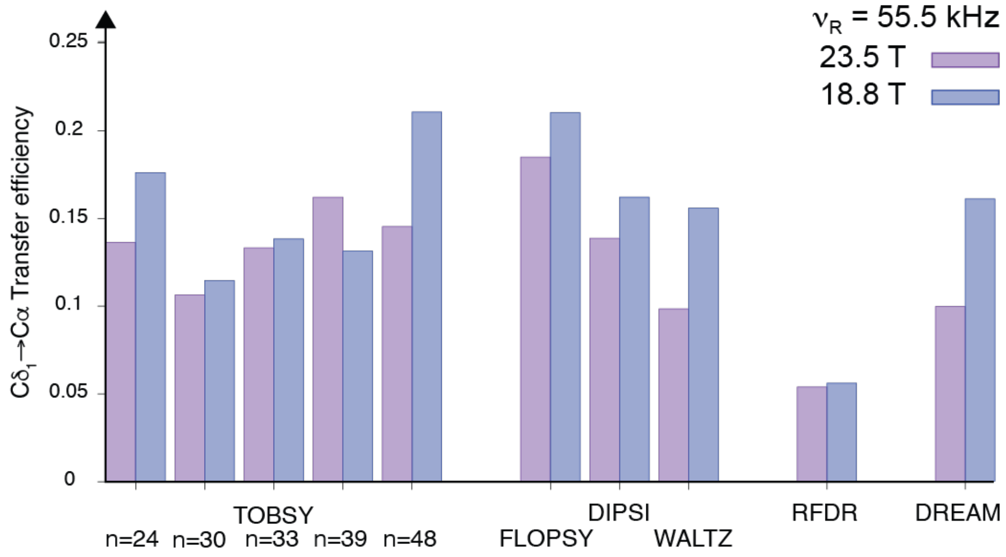

Supplementary Figure S12. Comparison of maximum attainable Cδ$_1 \rightarrow$ Cα transfer efficiency (at the optimal mixing times for each case) in a Leucine-like $^{13}C_6$ spin system simulated in SIMPSON for MAS frequency of $\nu_R = 55.5$ kHz for two magnetic field strengths: $B_0 = 18.8$ T (*blue* bars, right in each pair), and 23.5 T (*purple* bars, left in each pair). For DREAM, efficiency was evaluated at $\tau_{MIX} = 25$ ms (the largest mixing time sampled experimentally on fMLF).

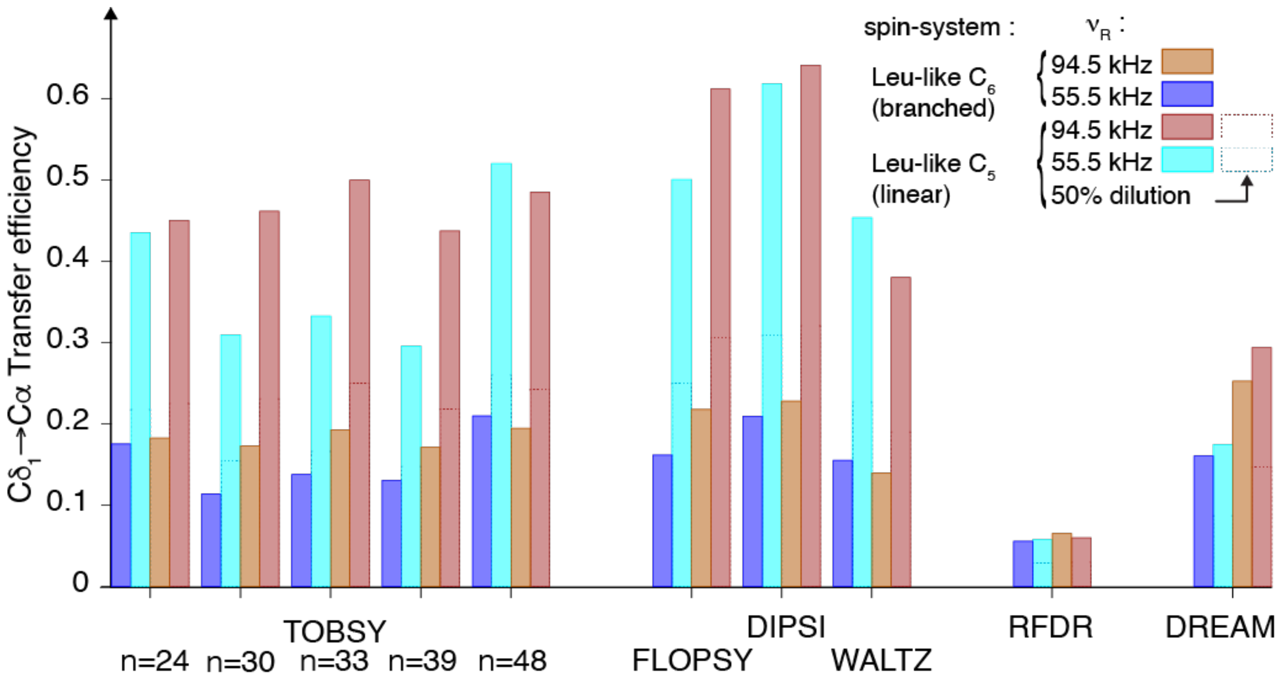

Supplementary Figure S13. Comparison of Cδ$_1 \rightarrow$ Cα transfer efficiency between Leucine-like *branched* six-^{13}C (*tan* bars) and *linear* five-^{13}C (*red* bars) spin systems, simulated in SIMPSON for ν_R = 100 kHz and 55.5 kHz and $B_0 = 18.8$ T field strength. Data for 55.5 kHz MAS is shown as *blue* and *cyan* bars, respectively. Efficiencies at the optimal mixing times for each case are shown. For a linear spin system ($^{13}C_5$) a sensitivity loss due to 50% spin dilution is indicated with dashed boxes.

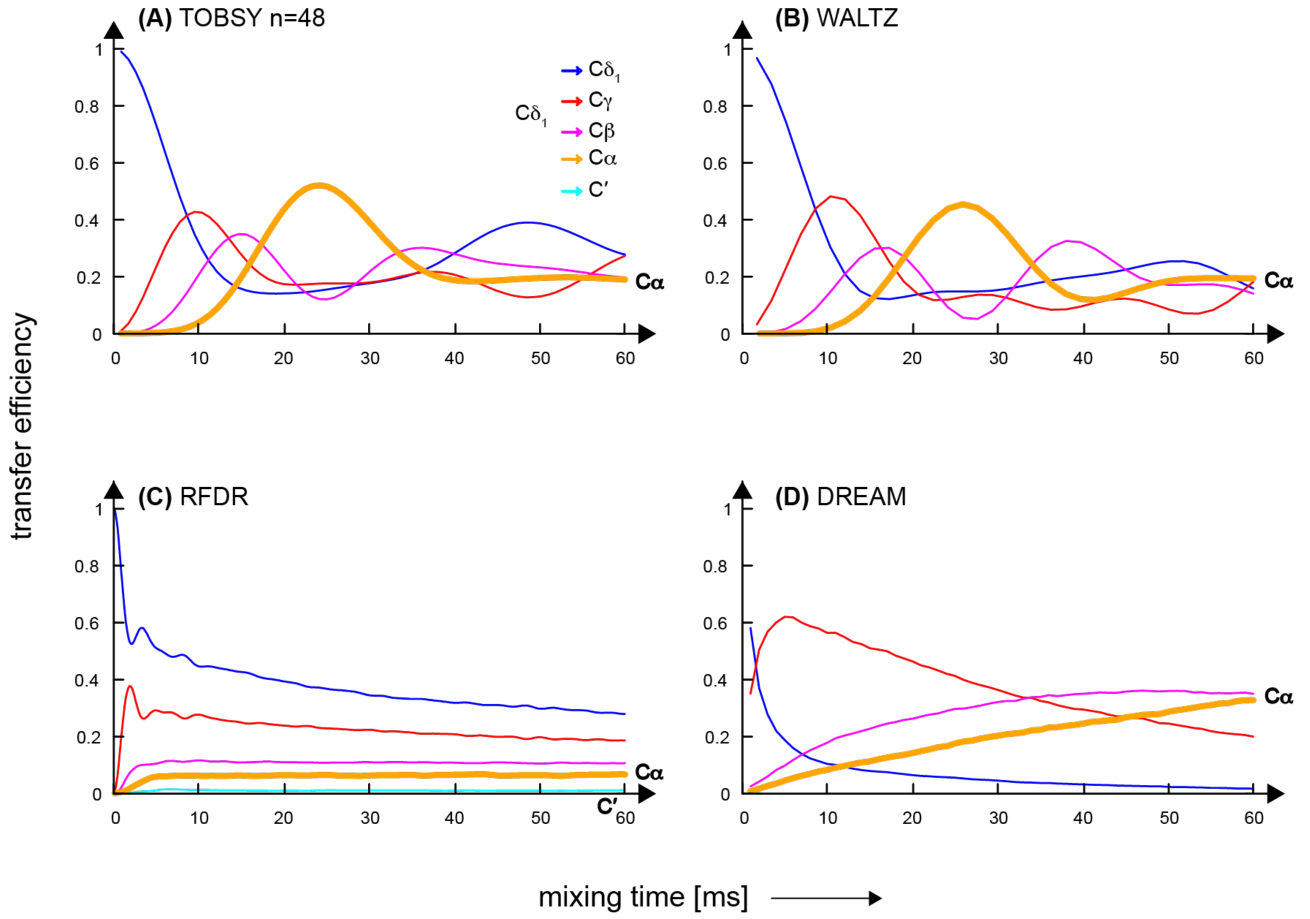

Supplementary Figure S14. Efficiency of coherence transfer from Cδ_1 to all other carbon spins in $^{13}C_5$ spin system (designed to resemble a leucine residue with a linear ^{13}C chain) in function of mixing time, simulated in SIMPSON for B_0 = 18.8 T field strength and ν_R = 55.5 kHz. The amplitude of oscillations for *J*-based transfers (A, B) is significantly increased with respect to analogous simulation for $^{13}C_6$ spin system shown in Supplementary Figure S6.

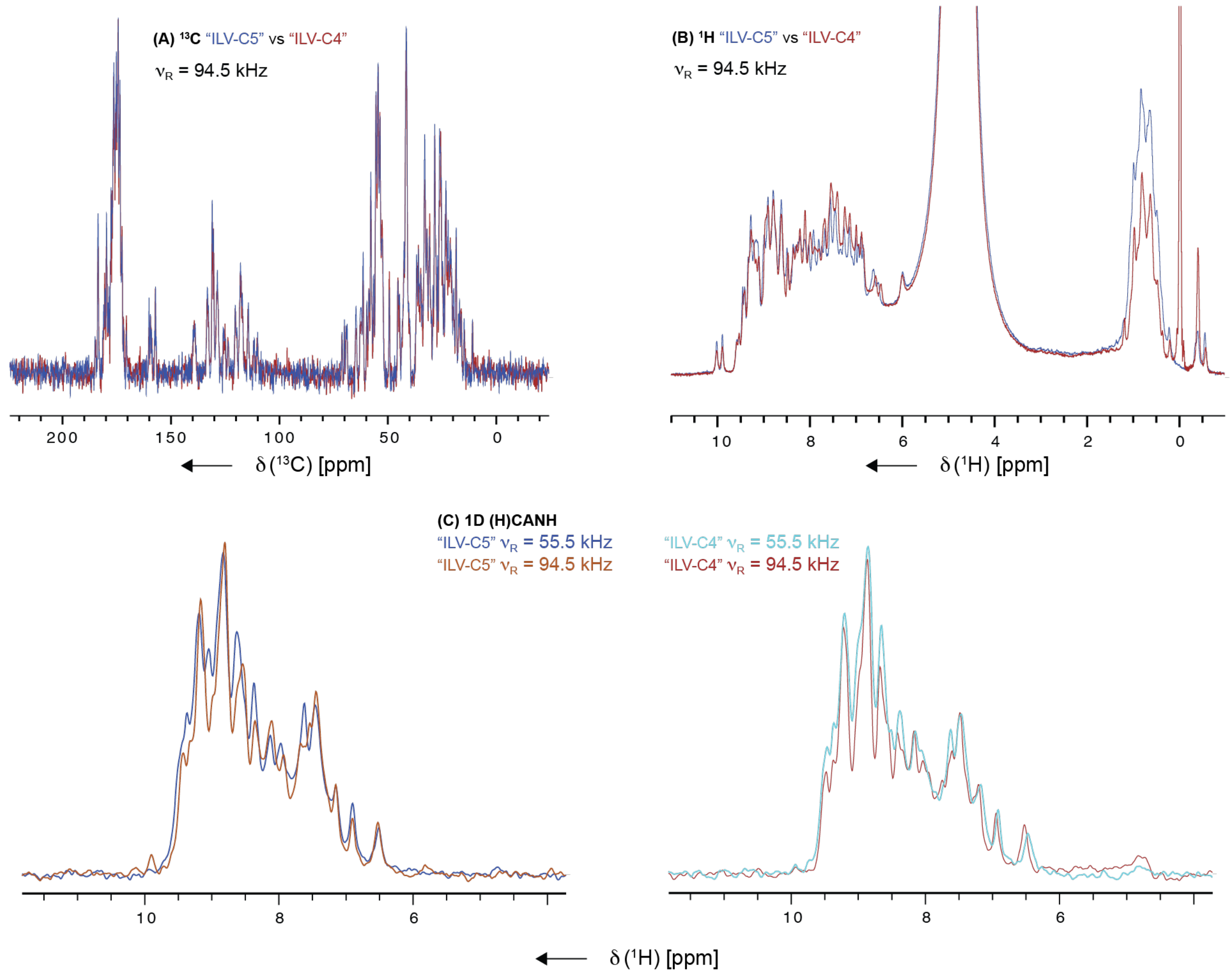

Supplementary Figure S15. Rough comparison of packing density and ^{1}H labelling of "ILV-C4" and "ILV-C5" SH3 samples. (A) 1D ^{13}C direct excitation spectra with an excessive recycling delay of 30 s (Bruker pulse program "hpdec"). (B) 1D ^{1}H direct excitation spectra, also at 94.5 kHz. (C) Efficiency of (H)CANH compared for "ILV-C4" and "ILV-C5"-labelled SH3 samples at 55.5 and 94.5 kHz MAS.

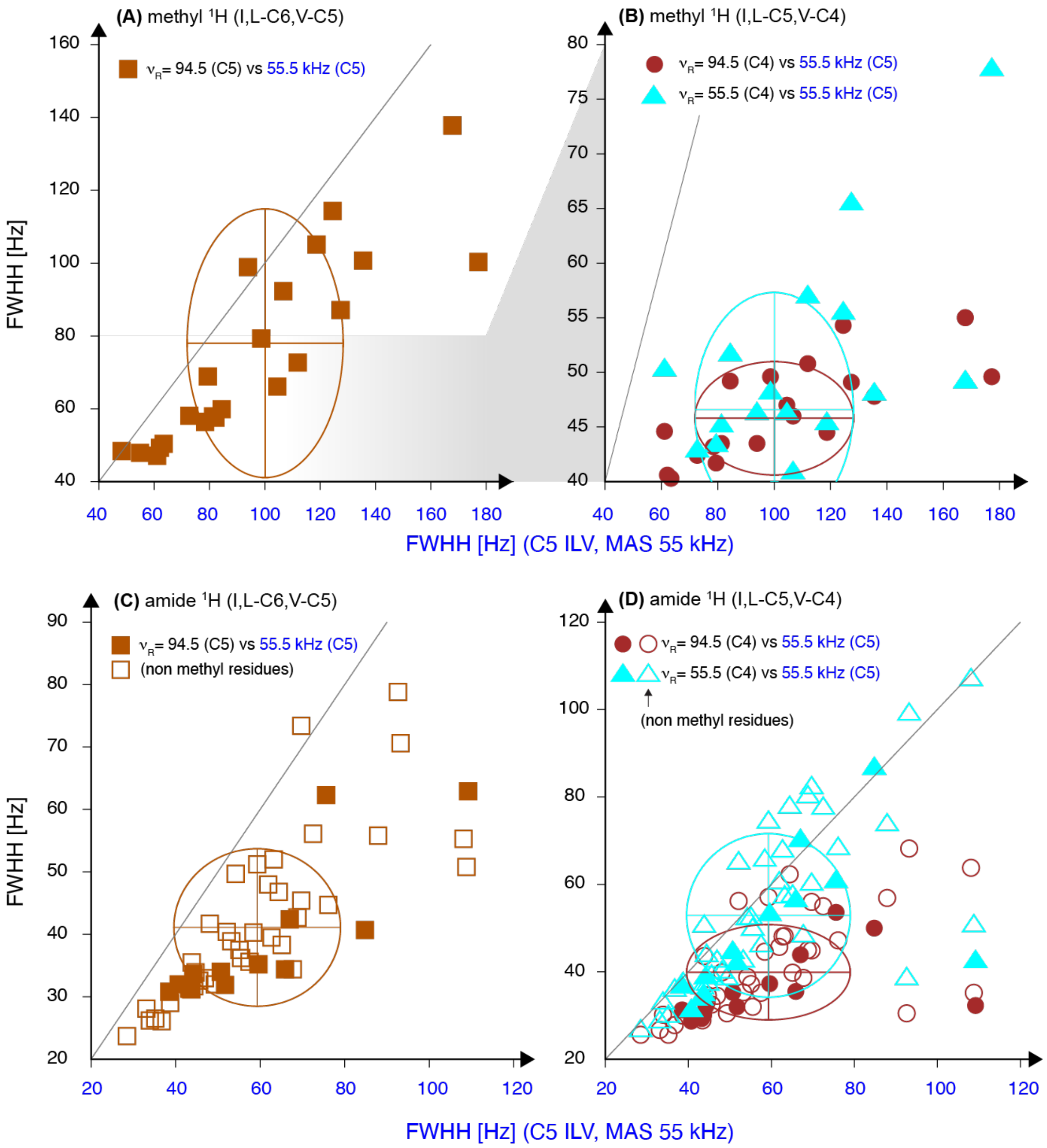

Supplementary Figure S16. Linewidths (full widths at half height) of (A, B) methyl and (C, D) backbone amide ¹H resonances observed for "ILV-C4" (*cyan* triangles for ν_R = 55.5 kHz and *red* circles for ν_R = 94.5 kHz) and "ILV-C5" (*tan* squares for ν_R = 94.5 kHz) SH3 samples, plotted with respect to values observed for "ILV-C5" sample ν_R = 55.5 kHz. Filled and open symbols in (C) and (D) correspond to amide ¹H resonances of ILV and other residues, respectively. Ellipses illustrate standard deviation of linewidths within given data set.

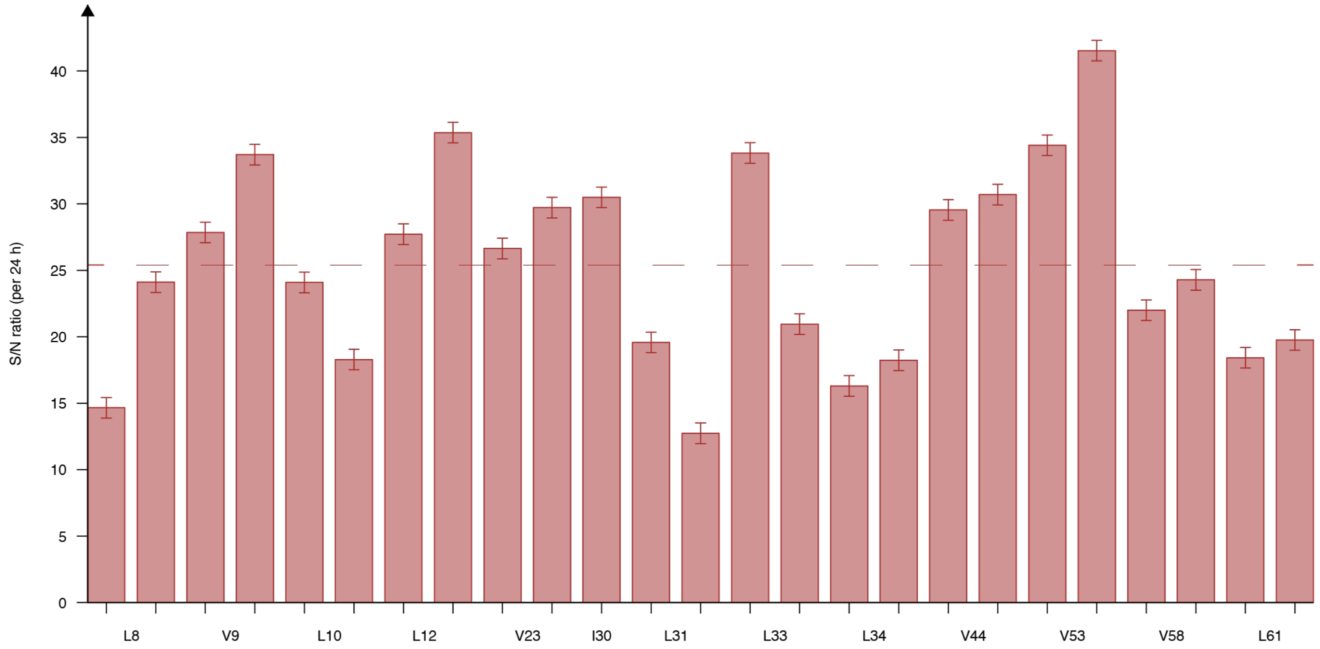

Supplementary Figure S17. Signal to noise RMSD ratios observed for individual cross-peaks in 4D HC(DIPSI)(CA)NH spectrum of "ILV-C4" SH3 protein sample at MAS frequency of 94.5 kHz and on an 18.8 T spectrometer, normalized to 24h experimental time.
